# Human expandable pancreatic progenitor–derived β cells ameliorate diabetes

**DOI:** 10.1126/sciadv.abk1826

**Published:** 2022-02-23

**Authors:** Xiaojie Ma, Yunkun Lu, Ziyu Zhou, Qin Li, Xi Chen, Weiyun Wang, Yan Jin, Zhensheng Hu, Guo Chen, Qian Deng, Weina Shang, Hao Wang, Hongxing Fu, Xiangwei He, Xin-Hua Feng, Saiyong Zhu

**Affiliations:** 1 The MOE Key Laboratory of Biosystems Homeostasis and Protection and Zhejiang Provincial Key Laboratory for Cancer Molecular Cell Biology, Life Sciences Institute, Zhejiang University, Hangzhou, China.; 2 Hangzhou Women’s Hospital, Prenatal Diagnosis Center, 369 Kunpeng Road, Hangzhou, China.; 3 Department of Pharmacy, Shulan (Hangzhou) Hospital Affiliated to Zhejiang Shuren University Shunlan International Medical College, 848 Dongxin Road, Hangzhou, China.; 4 Second Affiliated Hospital, School of Medicine, Zhejiang University, Hangzhou, China.

## Abstract

An unlimited source of human pancreatic β cells is in high demand. Even with recent advances in pancreatic differentiation from human pluripotent stem cells, major hurdles remain in large-scale and cost-effective production of functional β cells. Here, through chemical screening, we demonstrate that the bromodomain and extraterminal domain (BET) inhibitor I-BET151 can robustly promote the expansion of PDX1^+^NKX6.1^+^ pancreatic progenitors (PPs). These expandable PPs (ePPs) maintain pancreatic progenitor cell status in the long term and can efficiently differentiate into functional pancreatic β (ePP-β) cells. Notably, transplantation of ePP-β cells rapidly ameliorated diabetes in mice, suggesting strong potential for cell replacement therapy. Mechanistically, I-BET151 activates Notch signaling and promotes the expression of key PP-associated genes, underscoring the importance of epigenetic and transcriptional modulations for lineage-specific progenitor self-renewal. In summary, our studies achieve the long-term goal of robust expansion of PPs and represent a substantial step toward unlimited supplies of functional β cells for biomedical research and regenerative medicine.

## Teaser

Functional human β cells derived from expandable pancreatic progenitors show strong potential for regenerative medicine.

## INTRODUCTION

Diabetes mellitus represents a global health epidemic and affects millions of people worldwide. Islet transplantation holds great promises but is limited by shortage in supply of organ donors and immunosuppression issues associated with transplantation (*1*). Human pluripotent stem cells (hPSCs) can give rise to all cell types of the body (*2*, *3*). During the past two decades, stepwise differentiation protocols have been devised to guide the specification of hPSCs into definitive endoderm (DE), pancreatic progenitor (PP), endocrine precursor (EP), and pancreatic β–like cells (*4*–*17*). While these findings are highly encouraging, generating a large quantity of functional pancreatic β–like cells for disease modeling, drug screening, and cell-based therapy remains an extremely labor- and time-intensive process because of the multiple intermediate steps in directed differentiation.

A possible solution is to bypass the upstream steps starting with the hPSC source by initiating differentiation from renewable and expandable pancreatic progenitors (ePPs) that are developmentally more proximal to the β cells. However, robust expansion of human pancreatic progenitors has been challenging because the molecular mechanisms of human pancreatic progenitor self-renewal are poorly defined. In development, transcription factors (TFs) play an integral role, and the expression of key TFs is widely used to monitor the differentiation process and to evaluate cellular identity (*18*, *19*). During pancreatic development, expression of NKX6.1 follows that of PDX1 (*20*–*23*), and coexpression of PDX1 and NKX6.1 is widely used for defining and identifying pancreatic progenitors that can efficiently differentiate into functional pancreatic β–like cells (*5*–*8*). In recent years, several groups reported methods for culturing human endodermal derivatives at early developmental stages (*24*–*26*). In terms of pancreatic progenitors, Trott *et al.* (*27*) developed a culture condition that could expand PDX1-positive pancreatic progenitors, but unfortunately, these progenitors could not maintain the expression of NKX6.1. Pancreatic progenitors have also been expanded in three-dimensional (3D) culture (*28*–*30*). Ameri *et al.* (*31*) reported that genetic knockdown of cyclin-dependent kinase inhibitors *CDKN1A* and *CDKN2A* could increase the proliferation of glycoprotein 2–positive pancreatic progenitors, but here, application of the genetic methods may cause safety issues, which potentially limit its clinical usages. We previously demonstrated that pancreatic progenitors directly converted from human fibroblasts could be expanded in a chemically defined medium containing epithelial growth factor (EGF), basic fibroblast growth factor (bFGF), and A83-01 [a transforming growth factor–β (TGFβ) inhibitor], but the percentage of PDX1 and NKX6.1 double-positive cells was less than 20% (*32*). Whether this culture condition is suitable for human pancreatic progenitors directly differentiated from hPSCs has not been demonstrated so far. Thus, whether PDX1 and NKX6.1 double-positive pancreatic progenitors from hPSCs are competent for long-term expansion and, if yes, how to accomplish this goal are key questions in the field.

Synthetic chemical compounds provide useful tools to control cell fates and can also be used to decode the molecular mechanisms of biological processes (*33*, *34*). During the past decades, small molecules targeting specific signaling pathways are selected and applied on the basis of the knowledge learned from pancreatic developmental biology, and unbiased high-throughput chemical screening approaches have also been applied stepwise during the pancreatic differentiation process, resulting in the discovery of many effective small molecules for pancreatic differentiation (*5*, *35*–*38*). Small molecules have several advantages over the genetic methods, including that they are convenient to use, cost-effective, and can provide greater temporal control and be fine-tuned by varying their concentrations and combinations (*39*, *40*).

Here, we carried out a phenotypic chemical screen and identified that BET bromodomain inhibitor I-BET151 could significantly promote hPSC-derived pancreatic progenitor expansion. Next, we are able to uniformly capture and stably maintain ePPs from hPSCs. Furthermore, ePPs can be efficiently differentiated into functional pancreatic β–like cells (ePP-β cells). After transplantation, these ePP-β cells can rapidly ameliorate streptozotocin (STZ)–induced diabetes. Mechanistically, I-BET151 acts by promoting the expression of key pancreatic progenitor–associated genes and transcriptional network. Therefore, our findings establish a rapid, efficient, and readily controllable approach for the production of functional pancreatic β–like cells.

## RESULTS

### A chemical screen identified I-BET151 that can promote the expansion of human pancreatic progenitors

**Fig. 1.**
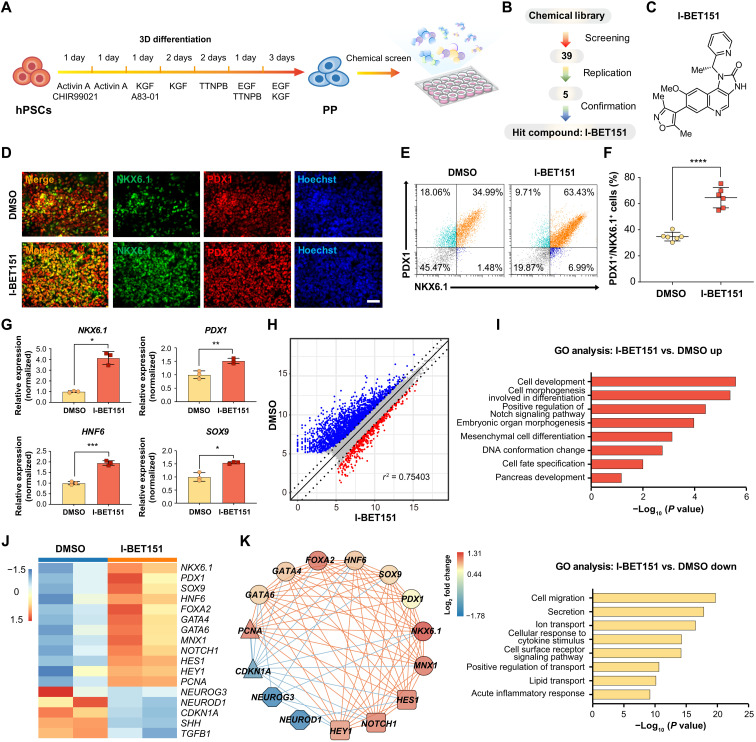
A chemical screen identified that I-BET151 can promote hPSC-derived pancreatic progenitor expansion. (**A**) Schematic representation of the pancreatic differentiation process from hPSCs to PPs and chemical screen for PP expansion. (**B**) Flowchart of the chemical screening process. After replication and confirmation, I-BET151 was found as the hit compound. (**C**) Chemical structure of I-BET151. (**D**) Representative immunofluorescent staining of PPs treated with or without I-BET151 for PDX1, NKX6.1, and nuclei. Scale bar, 100 μm. (**E** and **F**) Representative flow cytometry dot plots (E) and population percentages (F) of cells stained for PDX1 and NKX6.1. *N* = 6. (**G**) RT-qPCR analysis of *NKX6.1*, *PDX1*, *HNF6*, and *SOX9* gene expression in PPs treated with or without I-BET151. *N* = 3. (**H**) Volcano plot of differentially expressed genes (|log_2_FC| > 1, FDR < 0.05) in samples treated with I-BET151 versus dimethyl sulfoxide (DMSO). Red, up-regulated genes (620 genes); blue, down-regulated genes (2209 genes). *X* and *Y* axes represent log_2_(counts + 1). (**I**) GO terms of up-regulated and down-regulated genes in samples treated with I-BET151 versus DMSO. (**J**) Heatmap of differentially expressed genes in samples treated with I-BET151 versus DMSO. (**K**) I-BET151 strengthened the gene regulatory network of PPs (lines, the square of two genes’ expression’s Pearson correlation coefficient > 0.7; red lines, positive coexpression; blue lines, negative coexpression). Circle, PP marker genes; square, genes in Notch signaling pathway; triangle, cell cycle–related genes; octagon, EP markers. All data are expressed as means ± SD. Statistical significance was calculated using two-tailed Student’s *t* test, *P < 0.05, **P < 0.01, ***P < 0.001, and ****P < 0.0001.

We differentiated hPSCs into pancreatic progenitors (Fig. 1A) (*7*) and attempted to expand hPSC-derived pancreatic progenitors using chemically defined media. We applied real-time quantitative polymerase chain reaction (RT-qPCR) and immunostaining to monitor the pancreatic differentiation process (fig. S1, A to C). After pancreatic specification, fluorescence-activated cell sorting (FACS) results showed that the percentage of PDX1 and NKX6.1 double-positive cells was around 50 to 60% (fig. S1D). Next, we attempted to expand these hPSC-derived pancreatic progenitors, aiming at generating a large quantity of cells suitable for any downstream assays and applications. Unfortunately, the percentage of PDX1 and NKX6.1 double-positive cells was significantly reduced from ~60% to less than 20% after three to five passages (fig. S1, D and E), similar to earlier observations (*27*, *31*).

Next, we decided to carry out a chemical screen to identify small molecules that may support the expansion of PDX1 and NKX6.1 double-positive pancreatic progenitors (Fig. 1A). The detailed screening protocol is described in Materials and Methods. We used a basal medium (EF6 medium) containing EGF, bFGF, and 616452 (a TGFβ inhibitor) (*32*). The directed differentiated human pancreatic progenitors were passaged in basal medium containing EF6 for two to three passages to produce enough number of cells for downstream chemical screening. Briefly, cells were seeded into 24-well plates and then treated with small molecules from a chemical library, which contains bioactive small molecules mainly targeting epigenetic and signaling pathways (Fig. 1, A and B, and table S3). After 7-day treatments, we immunostained the cells for PDX1 and NKX6.1 and analyzed the percentage of PDX1 and NKX6.1 double-positive pancreatic progenitors using PE HCS screening platform (fig. S1F). We initially identified five candidates, and then we repeated the test above and confirmed that only the small molecule I-BET151 could significantly increase PDX1 and NKX6.1 double-positive pancreatic progenitors (Fig. 1B and fig. S1, G and H).

I-BET151 is a specific inhibitor of the acetyl-lysine binding reader bromodomain-containing proteins (BRDs) (Fig. 1C) (*41*). The effects of I-BET151 on pancreatic progenitor expansion were confirmed by detecting the coexpression of PDX1 and NKX6.1 using immunostaining and FACS (Fig. 1, D to F). Next, we evaluated the impact of different concentrations of I-BET151 on *NKX6.1* expression using RT-qPCR. We found that I-BET151 stimulated *NKX6.1* expression in a dose-dependent manner, and the optimal concentration was 1 μM (fig. S1I). We examined the expression level of other pancreatic progenitor maker genes *HNF6*, *SOX9*, *PDX1*, and *NKX6.1* and found that all of these marker genes were induced significantly upon I-BET151 treatment (Fig. 1G). Next, we assessed the possible changes in transcriptome induced by I-BET151 treatment using RNA sequencing (RNA-seq). The human pancreatic progenitors treated with and without I-BET151 were collected and subjected for RNA-seq. In total, 620 genes were up-regulated and 2209 genes were down-regulated after I-BET151 treatment [|log_2_fold change| > 1, false discovery rate (FDR) < 0.05] (Fig. 1H). Next, by Gene Ontology (GO) analysis, we found GO terms enriched among up-regulated genes included cell development, positive regulation of Notch signaling pathway, embryonic organ morphogenesis, cell fate specification, and pancreas development (Fig. 1I). The GO terms among down-regulated genes included cell migration, secretion, ion transport, lipid transport, and acute inflammatory response (Fig. 1I). In particular, a number of human pancreatic progenitor marker genes were transcriptionally up-regulated after I-BET151 treatment, including *NKX6.1*, *PDX1*, *SOX9*, *HNF6*, *FOXA2*, *GATA4*, *GATA6*, and *MNX1* (Fig. 1J). The expression of Notch signaling pathway genes was also significantly up-regulated, such as *NOTCH1*, *HEY1*, and *HES1* (Fig. 1J). In addition, the expression level of the proliferation-related gene *PCNA* also increased (Fig. 1J). On the other hand, the expression of the late-stage EP marker genes, such as *NEUROG3*, *NEUROD1*, and cell cycle inhibitor genes like *CDKN1A*, significantly decreased (Fig. 1J). Further gene coexpression network analysis demonstrated that the pancreatic progenitor marker genes and Notch signaling pathway genes were significantly positively coexpressed with each other while significantly negatively coexpressed with *NEUROG3*, *NEUROD1*, and *CDKN1A*, suggesting that I-BET151 robustly up-regulated and maintained the gene regulatory network of human pancreatic progenitors (Fig. 1K). Together, these data suggested that I-BET151 promoted pancreatic progenitor expansion.

Next, we tested the effects of other BET inhibitors on pancreatic progenitor expansion (*42*). We found that (+)-JQ1 could also promote PDX1 and NKX6.1 double-positive pancreatic progenitors, whereas the inactive stereoisomer (−)-JQ1 did not show any positive effect (fig. S2, A to D). In addition, RT-qPCR results showed that (+)-JQ1 could increase the expression levels of key pancreatic progenitor genes (fig. S2E). To test the target of I-BET151, we knocked down *BRD2*, *BRD3*, and *BRD4* genes by short hairpin RNAs (shRNAs), respectively (fig. S3A). During passage 1, shBRD4 could increase the expression of PP markers (fig. S3C), while shBRD2 and shBRD3 inhibited PP cell survival and decreased the expression of PP markers (fig. S3, B to D). After passaging, PPs transfected with shBRD4 could not proliferate in long term (fig. S3, E and F). These data suggested that shBRD4, rather than shBRD2 and shBRD3, could transiently mimic the effect of I-BET151. It is unclear why shRNAs against BRD4 show opposite effects in the long term. In addition, we applied dBET1, a potent BRD4 protein degrader based on proteolysis-targeting chimera technology (*43*), and confirmed that the presence of BRD4 protein is required for human pancreatic progenitor expansion (fig. S3, G to I). These results also highlight the unique advantages of unbiased chemical screening with a phenotypic and functional assay to identify potent small molecules that can be used to precisely control cell fate and status. Collectively, these results suggested that chemical inhibition of BET by I-BET151 promoted human pancreatic progenitor expansion.

### Robust and long-term expansion of hPSC-derived pancreatic progenitors

**Fig. 2.**
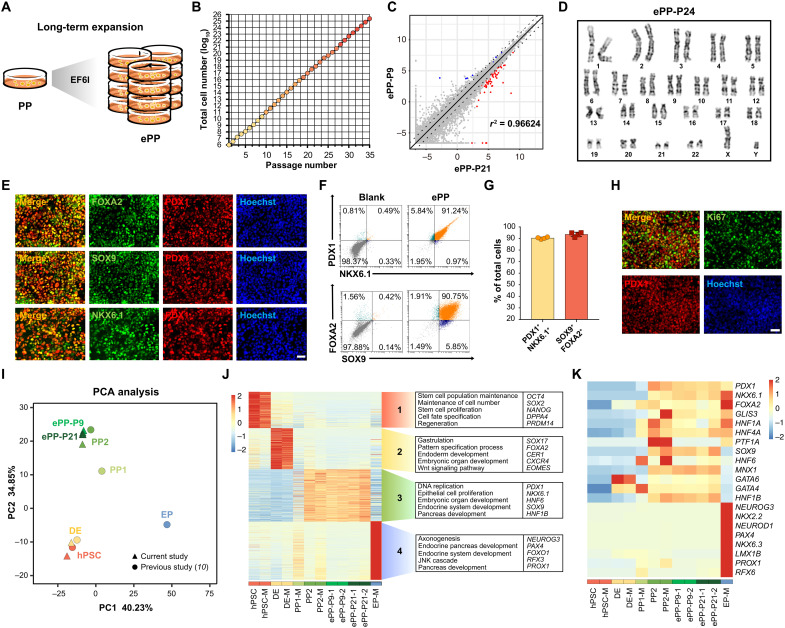
Long-term expansion and detailed characterization of ePPs. (**A**) Schematic of the long-term expansion of ePPs. (**B**) ePPs could be passaged more than 35 passages (*N* = 3). (**C**) Volcano plot comparing global gene expression patterns between ePP-P9 and ePP-P21. The red dots and blue dots respectively represent up-regulated (log_2_FC > 1) and down-regulated (log_2_FC < −1) differentially expressed genes in ePP-P21 versus ePP-P9 with statistical significance (FDR < 0.05). *X* and *Y* axes represent log_2_(TPM + 0.01). (**D**) Karyotype of ePP-P24 indicates the chromatin stability of ePPs after long-term expansion. (**E**) Representative immunofluorescent staining of ePPs for PDX1, FOXA2, SOX9, NKX6.1, and nuclei (Hoechst). Scale bar, 100 μm. (**F** and **G**) Representative flow cytometry dot plots (F) and population percentages (G) of PDX1^+^NKX6.1^+^ and SOX9^+^FOXA2^+^ cells demonstrated that ePPs were almost homogenous (*N* = 4). Data are expressed as means ± SD. (**H**) Representative immunofluorescent staining of ePPs for PDX1, Ki67, and nuclei. Scale bar, 100 μm. (**I**) Principal component analysis (PCA) of RNA-seq data. PP1, PDX1^+^ pancreatic progenitors; PP2, PDX1^+^NKX6.1^+^ pancreatic progenitors; ePP-P9, ePPs at passage 9; ePP-P21, ePPs at passage 21. Circle, data cited from a recently published paper from Melton laboratory (*10*); triangle, data of the current study. (**J**) Transcriptome analysis revealed different gene expression among hPSC, DE, PP1, PP2, ePP-P9, ePP-P21, and EP. M represents published data from Melton laboratory (*10*). Representative GO terms and genes are also shown. (**K**) Heatmap of PP- and EP-related marker genes in the samples shown in (I). Both early- and late-passage ePPs expressed PP-related marker genes including *PDX1*, *NKX6.1*, *SOX9*, *HNF6*, and *MNX1* but not EP-related marker genes such as *NEUROG3*, *NKX2.2*, and *NEUROD1*. JNK, c-Jun N-terminal kinase.

After repeated testing and multiple iterations of optimizing, we developed a defined culture condition (EF6I, which is EF6 medium plus I-BET151) for human pancreatic progenitor expansion (Fig. 2A). Using EF6I medium, we consistently established hPSC-derived ePPs that represent a new type of cells. Significantly, these ePPs cultured on feeder layers could be long-term expanded for at least 35 passages (Fig. 2B). Notably, these ePPs could be conveniently frozen and thawed, an important advantage enabling many downstream assays. The cellular identity of these ePPs was stable during expansion (fig. S4A). Both early-passage (passage 12) ePPs (ePP-P12) and late-passage (passage 24) ePPs (ePP-P24) demonstrated homogenous expression of PDX1 and NKX6.1 (fig. S4B). The RT-qPCR results demonstrated the stable expression of *HNF6*, *SOX9*, *PDX1*, and *NKX6.1* during long-term passaging (fig. S4C). Notably, global gene expression analysis using RNA-seq showed that the transcriptome of late-passage ePPs (ePP-P21) was very similar to that of early-passage ePPs (ePP-P9) (*r*^2^ = 0.96624), confirming the stability of ePPs at the transcriptome level (Fig. 2C). Karyotyping analysis demonstrated that these ePPs maintained a normal karyotype during long-term expansion (Fig. 2D and fig. S4D). The immunostaining results demonstrated the uniform expression of FOXA2, SOX9, PDX1, and NKX6.1 (Fig. 2E) within the cell population. Significantly, quantitative FACS analysis of PDX1 and NKX6.1 demonstrated almost homogenous expression in these ePPs, with about 90% of PDX1 and NKX6.1 double-positive cells, a level that is even higher than that of PP2 cells without expansion (Fig. 2, F and G). In addition, these ePPs were positive for the proliferative marker Ki67, an additional indication of their robust expansion capacity (Fig. 2H). We also derived PDX1 and NKX6.1 double-positive ePPs from human induced pluripotent stem cells (hiPSCs) and H9 human embryonic stem cells (hESCs), confirming the reproducibility of the expansion protocol (figs. S5 and S6, A to D).

To further evaluate the transcriptional profile of ePPs, we compared the transcriptomes of early- and late-passage ePPs (ePP-P9 and ePP-P21), hPSCs, DE, and PP2 by RNA-seq. We also compared our RNA-seq dataset with that from the recent work by Melton laboratory (hPSCs, DE, PP1, PP2, and EP, which were directly differentiated endoderm derivatives without expansion) (*10*). Principal component analysis (PCA) demonstrated that both early- and late-passage ePPs were transcriptionally similar to PP2 cells than to PP1 cells (Fig. 2I). With GO analysis, we found that genes specifically expressed in ePPs were related to DNA replication, epithelial cell proliferation, embryonic organ development, and pancreatic development, supporting their cellular identity of pancreatic progenitors (Fig. 2J). In particular, ePPs expressed key pancreatic progenitor genes, including *PDX1*, *NKX6.1*, *SOX9*, *HNF6*, and *MNX1*, but not EP genes, such as *NEUROG3*, *NKX2.2*, and *NEUROD1* (Fig. 2K). These results demonstrated that ePPs stably have a global transcriptional pattern similar to that of PP2 directly derived from hPSCs. Together, these results demonstrate that the long-term goal of robust expansion of human pancreatic progenitors is achieved, without any genetic manipulation.

### Efficient generation of functional pancreatic β–like cells from ePPs

**Fig. 3.**
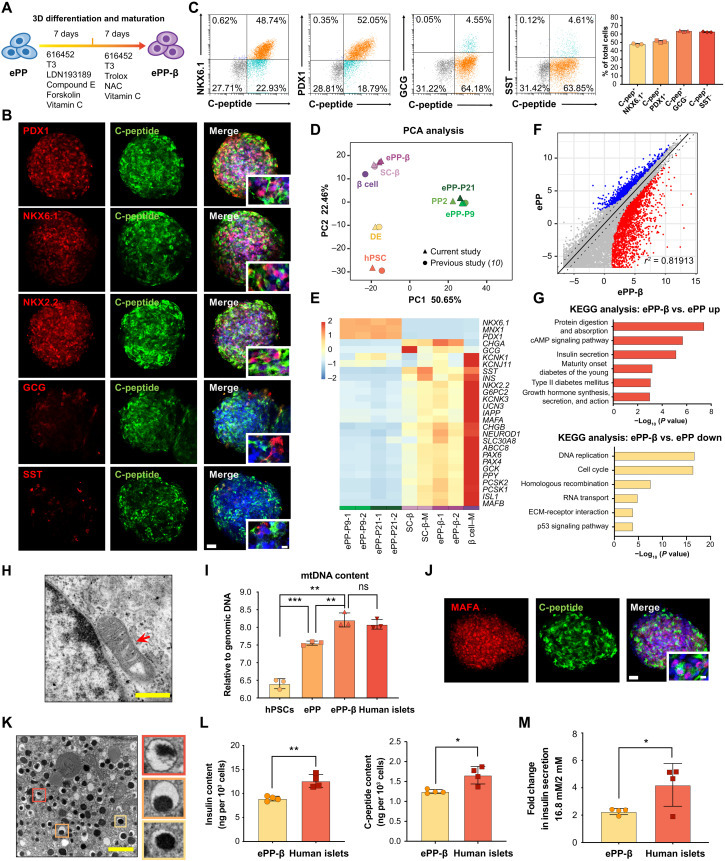
Efficient generation of functional ePP-β cells from ePPs. (**A**) Schematic indicating the differentiation of ePPs to ePP-β cells. (**B**) Representative immunofluorescence staining of ePP-β cells. Scale bars, 50 μm (low magnification) and 10 μm (high magnification). (**C**) FACS results (*N* = 3). (**D**) PCA of RNA-seq data. PP2, PDX1^+^NKX6.1^+^ pancreatic progenitors; ePP-P9, ePPs at passage 9; ePP-P21, ePPs at passage 21; ePP-β, ePP-derived β cells; SC-β, hPSC-derived β cells; β cell, human pancreatic islet β cells. Circle, data cited from a recently published paper (*10*); triangle, data of the current study. (**E**) The expression of β cell genes. M represents published data (*10*). (**F**) Volcano plot comparing global gene expression patterns. Significant up-regulated genes (log_2_FC > 1, FDR < 0.05) are shown in red (2173 genes), and significant down-regulated genes (log_2_FC < −1, FDR < 0.05) are shown in blue (1400 genes). *X* and *Y* axes represent log_2_(TPM + 0.01). (**G**) KEGG pathways enrichment. (**H**) Representative transmission electron micrographs of mitochondria in ePP-β cells. Scale bar, 500 nm. (**I**) Mitochondrial DNA (mtDNA) content. mtDNA content was measured by the ratio of mtDNA/nuclear DNA using qPCR for the mitochondrial 16*S* rRNA gene and the nuclear β*2 microglobulin* gene (*N* = 3). (**J**) Representative immunofluorescence staining of ePP-β cells. Scale bars, 50 μm (low magnification) and 10 μm (high magnification). (**K**) Representative transmission electron micrographs of insulin granules in ePP-β cells. Scale bar, 1 μm. (**L**) Total insulin and C-peptide content in ePP-β cells and human islets (*N* = 4). (**M**) GSIS analysis of ePP-β cells and human islets (*N* = 4). All data are expressed as means ± SD. Statistical significance was calculated using two-tailed Student’s *t* test: ns, *P* > 0.05; *P < 0.05; **P < 0.01; ***P < 0.001. cAMP, cyclic adenosine 3′,5′-monophosphate; ECM, extracellular matrix.

To address the question whether these hPSC-derived ePPs can further develop into functional pancreatic β–like cells (ePP-β cells), we applied an established protocol that can promote the differentiation of hPSC-derived pancreatic progenitors without expansion into insulin (INS)–producing pancreatic β–like cells (SC-β cells) (Fig. 3A). We efficiently generated INS^+^ ePP-β cells from both early- and late-passage ePPs (fig. S4E). Adding I-BET151 blocked the differentiation of ePPs into ePP-β cells, suggesting the stage-specific effects of I-BET151 (fig. S4F). By immunostaining, we found that these C-peptide–positive ePP-β cells coexpressed PDX1, NKX6.1, and NKX2.2 (Fig. 3B and figs. S5E and S6E). On the other hand, these cells rarely costained for other endocrine hormones, including glucagon (GCG) and somatostatin (SST) (Fig. 3B and figs. S5E and S6E). FACS analysis revealed the formation of about 40 to 60% monohormonal C-pep^+^GCG^−^, C-pep^+^SST^−^, C-pep^+^PDX1^+^, and C-pep^+^NKX6.1^+^ ePP-β cells (Fig. 3C). We observed comparable differentiation capacity of ePPs from hESCs and hiPSCs (fig. S5, E and F). Thus, long-term expansion does not alter the β cell differentiation capacity of ePPs.

To evaluate the transcriptional profile of ePP-β cells, we compared the transcriptomes of ePP-β cells, early- and late-passage ePPs (ePP-P9 and ePP-P21), hPSCs, DE, and PP2 by RNA-seq and also with the RNA-seq dataset (hPSCs, DE, PP2, SC-β, and β cells) from the recent work by Melton laboratory (*10*). PCA demonstrated that ePP-β cells were transcriptionally similar to SC-β cells (Fig. 3D). Compared to ePPs, many β cell genes were significantly up-regulated in ePP-β cells, including *INS*, *IAPP*, *SLC30A8*, *PCSK1*, *GCK*, and *ABCC8* (Fig. 3E). Further analysis demonstrated that 2713 genes were up-regulated and 1400 genes were down-regulated after differentiation (ePP-β cells versus ePPs) (Fig. 3F). Up-regulated Kyoto Encyclopedia of Genes and Genomes (KEGG) terms included protein digestion and absorption, cyclic adenosine 3′,5′-monophosphate signaling pathway, insulin secretion, maturity onset diabetes of the young, type II diabetes mellitus, and growth hormone synthesis, secretion, and action (Fig. 3G). Down-regulated KEGG terms included DNA replication, cell cycle, homologous recombination, RNA transport, extracellular matrix–receptor interaction, and p53 signaling pathway (Fig. 3G). These results suggest that ePP-β cells are transcriptionally similar to SC-β cells.

To further evaluate the functional capacity of these ePP-β cells, we did the ultrastructural analysis for mitochondria and insulin production using transmission electron microscopy (TEM). We observed the morphology of mature mitochondria and detected the up-regulation of mitochondrial DNA (mtDNA) content in ePP-β cells in comparison to hPSCs and ePPs and comparable to that of human islets (Fig. 3, H and I). Significantly, immunostaining data showed that ePP-β cells expressed a high level of mature β-cell marker MAFA (Fig. 3J). In addition, the TEM result revealed that these ePP-β cells contained numerous typical insulin granules exhibiting electron dense cores (Fig. 3K). In addition, we examined the insulin and C-peptide content using enzyme-linked immunosorbent assay (ELISA) and found that ePP-β cells contained 1.248 ± 0.025 ng of C-peptide per 1000 cells and 8.93 ± 0.2 ng of insulin per 1000 cells (Fig. 3L and fig. S5G). These values are comparable to those of SC-β cells and human islets (*12*). Moreover, a key feature of a functional pancreatic β cell is its ability to release insulin upon glucose stimulation. To test this for ePP-β cells, we performed the experiments of glucose-stimulated insulin secretion (GSIS). The stimulation index, as calculated by the ratio of insulin secreted in high glucose (16.8 mM) to that in low glucose (2 mM), was around 2 for hESC-derived ePP-β cells and 1.5 to 1.6 for hiPSC-derived ePP-β cells (Fig. 3M and figs. S5H and S6F). These values are comparable to those in SC-β cells and islet β cells (*6*, *7*, *11*, *12*). Thus, ePP-β cells were capable of sensing repeated low- and high-glucose impulses. Together, our results illustrate that ePP-β cells are functional in vitro.

### Human ePP-β cells can rapidly ameliorate diabetes

**Fig. 4.**
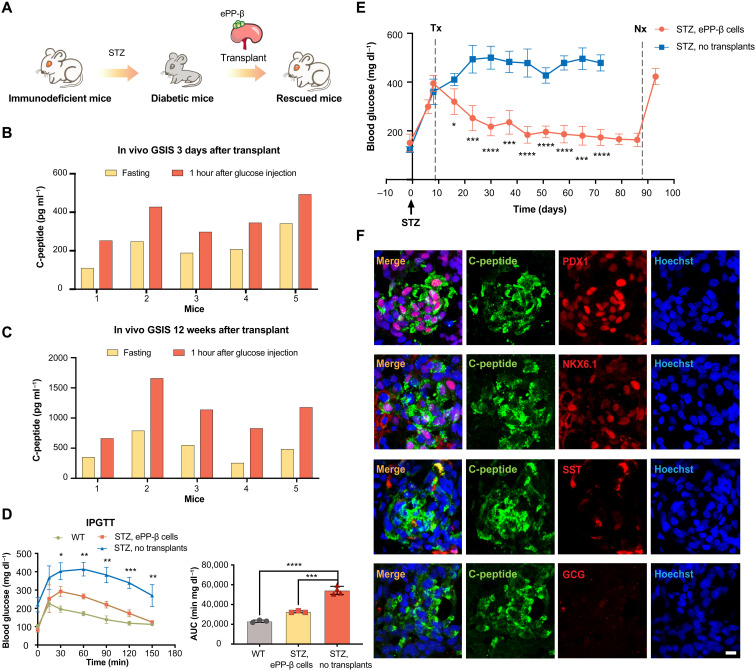
ePP-β cells can rapidly ameliorate diabetes. (**A**) Schematic showing transplantation of ePP-β cells into immunodeficient diabetic mice. (**B** and **C**) In vivo glucose challenge test 3 days (B) and 12 weeks (C) after transplantation. The levels of human C-peptide in mouse serum were measured after overnight fasting (yellow) and 1 hour after an intraperitoneal glucose injection (orange). The *x* axis represents the number of individual animals (*N* = 5). (**D**) Intraperitoneal glucose tolerance test (IPGTT) was performed 30 days after transplantation with ePP-β cells (*N* = 3), nontransplanted diabetic SCID beige mice (*N* = 4), and nontransplanted wild-type (WT) SCID beige mice (*N* = 3). Area under the curve (AUC; left) was determined for each group (right). (**E**) Blood glucose level of STZ-treated control mice (no transplant, blue) and mice transplanted with ePP-β cells (orange). Tx, transplantation; Nx, nephrectomy. Control group (*N* = 5) and experimental group (*N* = 4). (**F**) Representative immunofluorescent images of ePP-β grafts stained with C-peptide, NKX6.1, PDX1, SST, GCG, and nuclei. Scale bar, 10 μm. All data are expressed as means ± SD. Statistical significance was calculated using two-tailed Student’s *t* test, *P < 0.05, **P < 0.01, ***P < 0.001, and ****P < 0.0001.

Functional pancreatic β cells are capable of regulating glucose levels in surrogate animals. To evaluate the in vivo functions of ePP-β cells, we transplanted them into immunodeficient mice (Fig. 4A). Notably, we detected significant GSIS in ePP-β–transplanted mice as early as 3 days after transplantation (Fig. 4B), and the GSIS capacity was maintained at 12 weeks after transplantation (Fig. 4C). Encouraged by this result, we transplanted ePP-β cells under the kidney capsule of mice rendered diabetic through treatment with STZ. We performed a glucose tolerance test on mice transplanted with ePP-β cells 30 days after transplantation. Mice transplanted with ePP-β cells were significantly more glucose tolerant and also cleared glucose faster than mice without transplantation of ePP-β cells (Fig. 4D). Notably, for the blood glucose levels, mice transplanted with ePP-β cells significantly reduced in 2 weeks after transplantation, and these mice became and maintained euglycemia after 3 weeks (Fig. 4E). About 12 weeks later, ePP-β grafts were removed by nephrectomy, and mice rapidly developed diabetes, demonstrating that ePP-β cells indeed controlled glucose levels in STZ-treated mice (Fig. 4E). Immunofluorescence analysis of ePP-β grafts revealed that C-peptide–positive cells coexpressed critical β cell TFs PDX1 and NKX6.1 but did not exhibit expression of the other endocrine hormones GCG and SST (Fig. 4F). Collectively, these results demonstrate that ePP-β cells maintain their differentiated phenotype and remain glucose responsive after engraftment in vivo and can rapidly ameliorate diabetes, which highlight their potential therapeutic values.

### Molecular mechanisms of I-BET151 on promoting human pancreatic progenitor expansion

**Fig. 5.**
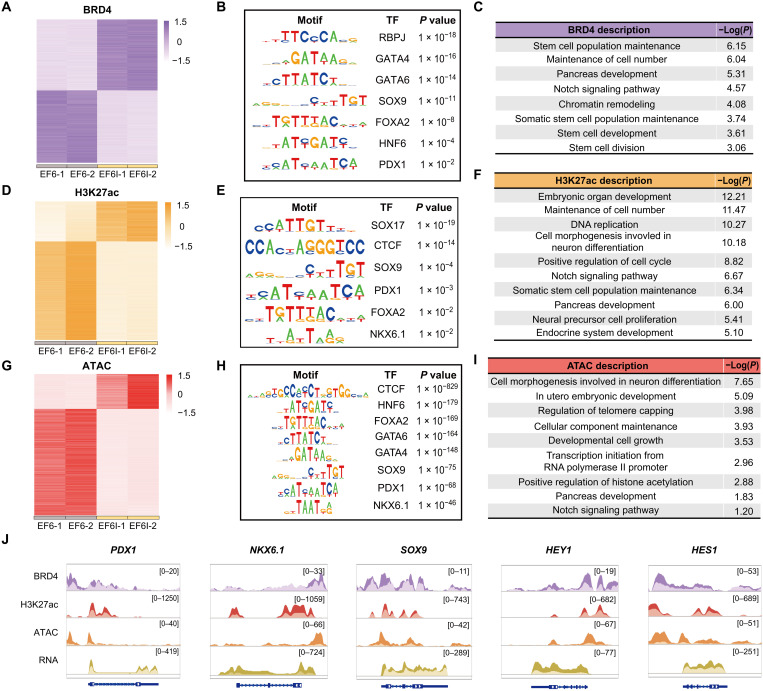
Molecular mechanisms of I-BET151. (**A**) Differential BRD4-binding peaks in PP cells cultured in EF6 or EF6I medium. (**B**) Transcription factor (TF) binding motifs enriched at up-regulated BRD4-binding peaks induced by I-BET151. (**C**) GO categories of BRD4-binding peaks specifically enhanced by I-BET151. (**D**) Differential H3K27ac peaks in PP cells cultured in EF6 or EF6I medium. (**E**) TF binding motifs enriched at up-regulated H3K27ac peaks induced by I-BET151. (**F**) GO categories of H3K27ac peaks specifically enhanced by I-BET151. (**G**) Differential ATAC peaks in PP cells cultured in EF6 or EF6I medium. (**H**) TF binding motifs enriched at up-regulated ATAC peaks induced by I-BET151. (**I**) GO categories of enriched, more accessible genes induced by I-BET151. (**J**) Browser tracks showing BRD4-binding peaks, H3K27ac peaks, ATAC peaks, and RNA abundance at *PDX1*, *NKX6.1*, *SOX9*, *HES1*, and *HEY1* loci 7 days after I-BET151 treatment. EF6 group, peaks in brighter colors; EF6I group, peaks in darker colors.

To study the binding patterns of BRD4 after I-BET151 treatment, we applied cleavage under targets and tagmentation (CUT&Tag) (fig. S7, A to C) and observed I-BET151–induced changes of BRD4-binding peaks (Fig. 5A and fig. S7E). TF binding motif enrichment analysis of I-BET151–induced BRD4 peaks identified RBPJ, GATA4, GATA6, SOX9, FOXA2, HNF6, and PDX1 as the top-enriched TF binding motifs (Fig. 5B). GO terms of induced peak-related genes were associated with stem cell population maintenance, pancreatic development, Notch signaling pathway, and stem cell division (Fig. 5C). In particular, BRD4 binding signals at *PDX1*, *NKX6.1*, *SOX9*, *HEY1*, and *HES1* loci were all increased upon I-BET151 treatment (Fig. 5J). These observations suggested that I-BET151 modulated the genome-wide binding patterns of BRD4, consisting with changes in transcriptome.

To further investigate the regulations at genome-wide enhancer landscape and chromatin accessibility, we performed chromatin immunoprecipitation combined with sequencing (ChIP-seq) of H3K27ac and assay for transposase-accessible chromatin using sequencing (ATAC-seq) (fig. S7, A and B). We observed global increases of H3K27 acetylation and chromatin accessibility at I-BET151–induced genes (fig. S7, C, D, F, and G). Then, we further analyzed I-BET151–induced H3K27ac peaks and induced ATAC peaks (Fig. 5, D and G). TF binding motif enrichment analysis of I-BET151–induced H3K27ac peaks identified SOX17, CTCF, SOX9, PDX1, and FOXA2 as the top-enriched TF binding motifs (Fig. 5E). In addition, H3K27 acetylation level increased at *PDX1*, *NKX6.1*, *SOX9*, *HEY1*, and *HES1* loci upon I-BET151 treatment (Fig. 5J). For I-BET151–induced ATAC peaks, we identified CTCF, HNF6, FOXA2, GATA6, GATA4, SOX9, PDX1, and NKX6.1 as the top-enriched TF binding motifs (Fig. 5H). In particular, chromatic accessibility increased at *PDX1*, *NKX6.1*, *SOX9*, *HEY1*, and *HES1* loci upon I-BET151 treatment, highly consistent with the transcriptional up-regulation of these genes (Fig. 5J). In addition, GO terms of induced peak-related genes were associated with embryonic development, DNA replication, Notch signaling pathway, and pancreatic development (Fig. 5, F and I). Cumulatively, these results suggested that I-BET151 could modulate the enhancer landscape, increase the chromatin accessibility, up-regulate the transcription of key pancreatic progenitor genes, and stabilize the gene regulatory network of human pancreatic progenitors.

## DISCUSSION

During pancreatic development, multipotent pancreatic progenitor cells with inherent proliferative capacity and coexpressing PDX1 and NKX6.1 are responsible for the proper growth of pancreas. Developmentally more committed cells, such as the NGN3-positive EPs and INS-positive β cells, are with limited to no proliferative capacity.

Therefore, expansion of pancreatic progenitors that are developmentally proximal to the β cells is a promising approach for producing an unlimited number of pancreatic β cells. Here, through unbiased phenotype-based chemical screening, we identified small molecules that could enhance NKX6.1, SOX9, and HNF6 expression but reduce NGN3, NEUROD1, and NKX2.2 expression, indicating the stabilization of pancreatic progenitor cell state. Further in-depth analysis of these PDX1 and NKX6.1 double-positive ePPs will provide invaluable information about the mechanisms underlying pancreatic progenitor self-renewal in a human setting that has remained hard to study because of the limited supply of embryonic samples at the relevant stages.

During development in vivo, the proliferation of pancreatic progenitor cells is promoted by factors secreted by the surrounding mesenchymal tissue (*44*). Here, our studies showed that in vitro differentiated human pancreatic progenitor cells (derived from hPSCs) could be long-term expanded using I-BET151 in a chemically defined condition. We found that I-BET151 could transcriptionally up-regulate key genes of pancreatic progenitors. Initially, I-BET151 was identified as an inhibitor of cell proliferation and cancer development (*41*), and I-BET151 treatment blocked ESC self-renewal (*45*). Counterintuitively, we found that I-BET151 could promote human pancreatic progenitor expansion, which highlights the context-dependent effects of BET inhibition. Recently, the positive effects of BET inhibition on transcriptional regulation have also been reported in other biological processes, including autophagy (*46*), HIV-1 latency (*47*), and naïve pluripotency (*48*). Thus, chromatin modification–based transcriptional regulation is context-dependent. Further investigations on how I-BET151 promotes human pancreatic progenitor expansion can provide better understanding of epigenetic modulation, transcriptional regulation, and cell fate decision.

It is very encouraging that these hPSC-derived ePPs can be efficiently differentiated into functional ePP-β cells. These ePPs and ePP-β cells provide new opportunities for investigation in developmental biology, disease modeling, and large-scale drug screening. For example, patient-specific ePPs and ePP-β cells can be efficiently generated, with the hope to bypass the immunosuppression complications associated with transplantation. Furthermore, genome editing tools can be applied to test variants identified by genome-wide associated studies (*49*, *50*). These ePPs and ePP-β cells will provide a new and readily controllable platform for drug screening and for studying human metabolism and diabetes (*51*).

As limited supplies of donated islets have severely limited islet transplantation, the strategy that we describe here may address this limitation and present an opportunity for cell-based therapy. These hPSC-derived ePPs can be efficiently differentiated into functional ePP-β cells with high quality. Promisingly, with the capacity of robust expansion free of foreign genetic material and further efficient differentiation into functional pancreatic β–like cells, the established procedure should be upscalable for functional pancreatic β cell manufacture at large quantity. In summary, our work on the robust expansion of PDX1 and NKX6.1 double-positive human pancreatic progenitors represents a notable step toward providing unlimited functional human pancreatic β cells that are of great interest for biomedical research and regenerative medicine.

## MATERIALS AND METHODS

### Cell culture

Human embryonic kidney (HEK) 293T cells were maintained in Dulbecco’s modified Eagle’s medium (DMEM) (Life Technologies) containing 10% fetal bovine serum (FBS) (Gibco) and 1× penicillin/streptomycin (Life Technologies). HEK293T cells were bought from the American Type Culture Collection (ATCC) (CRL-3216).

Undifferentiated hESCs and hiPSCs were cultured in hPSC medium: DMEM/F12 (Life Technologies) supplemented with 20% KnockOut serum replacement (Life Technologies), 1× nonessential amino acids (Life Technologies), 1× penicillin/streptomycin, 0.055 mM 2-mercaptoethanol (Sigma-Aldrich), and bFGF (10 ng ml^−1^; Peprotech). hPSCs were maintained on CF1 feeder cells in a humidified incubator at 37°C and 5% CO_2_. hPSCs were passaged by Accutase (Life Technologies) as 1:3 to 1:6 every 3 to 6 days. Thiazovivin (0.5 μM; TargetMol) was used during the first 24 hours when passaging or thawing cells. MEL1 INS^GFP/W^ hESC line was a gift from E. G. Stanley and A. Elefanty. H9 hESCs were purchased from Wicell Research Institute. hiPSCs were produced by reprogramming human fibroblasts that were purchased from ATCC (CRL-2097) (*49*, *52*). Mycoplasma contamination was routinely detected using the TransDetect PCR Mycoplasma Detection Kit (TransGen Biotech). Institutional Animal Care and Use Committee guidelines were followed with human or animal subjects.

### Cell differentiation

Cells were differentiated according to a previously described protocol (*7*). hPSCs were seeded into 10-cm dishes and cultured for 4 days before differentiation. The medium was changed every day. Then, hPSCs were washed by Dulbecco’s phosphate-buffered saline (DPBS; Life Technologies) followed by dissociation into single cells using Accutase. The cells were counted and seeded at 5.5 × 10^6^ cells per well of low-attached six-well plates (Corning) in 5.5 ml of hPSC medium supplemented with activin A (10 ng ml^−1^; Peprotech) and heregulin B (10 ng ml^−1^; Peprotech). The plates were plated on a shaker at 100 rpm in an incubator to promote cell aggregate formation. The media were changed every day. The differentiation media are as follows: day 1: RPMI (Life Technologies) including 1:5000 ITS-X (Gibco), 0.2% FBS, activin A (100 ng ml^−1^), and 3 μM CHIR99021 (TargetMol); day 2: RPMI containing 0.2% FBS, 1:2000 ITS-X, and activin A (100 ng ml^−1^); day 3: RPMI containing 0.2% FBS, 1:1000 ITS-X, KGF (25 ng ml^−1^; Peprotech), and 2.5 μM A83-01 (TargetMol); days 4 to 5: RPMI containing 0.4% FBS, 1:1000 ITS-X, and KGF (25 ng ml^−1^); days 6 to 7: DMEM containing 1× B27 (Gibco) and 3 nM TTNPB (Sigma-Aldrich); day 8: DMEM containing 1× B27, 3 nM TTNPB, and EGF (50 ng ml^−1^; Peprotech); days 9 to 11: DMEM containing 1× B27, KGF (50 ng ml^−1^), and EGF (50 ng ml^−1^); days 12 to 18 (R6 medium): DMEM containing 1× B27, 0.05% bovine serum albumin (BSA; Yeasen), 10 μM zinc sulfate (Sigma-Aldrich), heparin (10 μg ml^−1^; Sigma-Aldrich), 10 μM 616452 (TargetMol), 1 μM T3 (Sigma-Aldrich), 0.2 μM LDN-193189 (Tocris), 0.2 μM compound E (MedChem Express), 0.5 mM vitamin C, and 10 μM forskolin (TargetMol); days 19 to 24 (R7 medium): DMEM containing 1× B27, 0.05% BSA, 10 μM zinc sulfate, heparin (10 μg ml^−1^), 10 μM 616452, 1 μM T3, 1 mM *N*-acetyl cysteine (Sigma-Aldrich), 1 μM Trolox (Sigma-Aldrich), and 0.5 mM vitamin C.

### ePP culture

ePPs were cultured on feeder cells in EF6I medium: DMEM containing 1× B27, EGF (50 ng ml^−1^), bFGF (10 ng ml^−1^), 10 μM 616452, and 1 μM I-BET151 (APExBIO). ePPs were passaged by Accutase (Life Technologies) at a ratio of 1:3 every 3 to 5 days. Routinely, 0.5 μM thiazovivin was used during the first 24 hours when passaging or thawing cells.

### Chemical screening

A chemical library containing about 203 small molecules, listed in table S3, was used for screening. PP cells cultured in basal medium [DMEM containing 1× B27, EGF (50 ng ml^−1^), bFGF (10 ng ml^−1^), and 10 μM 616452] were seeded into 24-well plates and administrated with different small molecules for 7 days. The culture media were changed every 3 days. Then, PP cells were immunostained with anti-PDX1 and anti-NKX6.1 antibodies. In addition, images were generated using the Operetta CLS high-content analysis system (PerkinElmer) and analyzed by the software of Operetta CLS high-content analysis system.

### Immunostaining

Cells were stained according to a previous protocol (*49*). In brief, cells were fixed with 4% paraformaldehyde (PFA) for 10 to 15 min at room temperature, washed with PBST buffer three times, and blocked in blocking buffer for 1 hour at room temperature followed by incubating with the primary antibody at 4°C overnight. Then, secondary antibodies were used at 1:2000 dilution and incubated for 1 hour at room temperature. Detailed primary and secondary antibodies are listed in table S1. Last, cells were stained with Hoechst at 1:5000 dilution to mark nuclei.

### Flow cytometry

Cells were dissociated into single cells using Accutase followed by washing with PBS buffer. Then, the cells were fixed by 4% PFA for 30 min and washed with PBST three to five times at 1500 rpm, 5 min per time. Thereafter, cells were blocked with blocking buffer and incubated with primary antibodies at 4°C overnight. After washing using PBST for three times, cells were incubated with secondary antibodies at room temperature for 1 hour. All the antibodies are detailed in table S1. FACS data were acquired by Beckman CytoFlex (Beckman Culture) and analyzed by CytExpert software.

### Real-time quantitative PCR

Total RNA was extracted and purified using the Quick-RNA MiniPrep Kit (ZYMO) followed by conversion into complementary DNA (cDNA) using PrimeScript RT Master Mix (Takara). RT-qPCR was performed using the TB Green Premix Ex Taq II Kit (Takara) on a CFX Connect Real-Time system (Bio-Rad). Primer sequences are listed in table S2.

### shRNA studies

Lentiviral vectors (pLKO.1) carrying independent *BRD2*-, *BRD3*-, and *BRD4*-targeting or control shRNAs (sequences are shown in table S1) were transfected into HEK293T cells maintained in DMEM medium using Lipofectamine 3000 Transfection Reagent (Invitrogen). Viruses were collected and filtered 48 and 72 hours after transfection.

For lentiviral infection, 60 to 70% confluent PPs were incubated with viruses and basal medium (EF6) as 1:1. In addition, Polybrene infection reagent (Santa Cruz Biotechnology) was added at 5 μg ml^−1^. After 4 hours, basal medium was used to culture cells for 1 week. During this week, medium was changed every 3 days.

For infected cell collection, PPs with different shRNAs were dissociated by incubation with Accutase for 1 to 3 min at 37°C. Then, cell samples were collected for RNA extraction and RT-qPCR analysis.

### Western blotting analysis

Lysis buffer (Beyotime) supplemented with 1% phenylmethylsulfonyl fluoride (Beyotime) was used to extract total protein from cells. Cell extracts were centrifuged at 12,000*g* for 15 min, and the supernatant was collected. Cell lysates were resolved on 8% acrylamide gradient SDS–polyacrylamide gel electrophoresis gels and transferred to polyvinylidene difluoride membranes (Millipore). The membranes were blocked with 5% nonfat milk in tris-buffered saline containing 0.1% Tween 20 for 1 hour, then incubated with anti-BRD4 (1:1000 dilution; Abcam) and anti–β-actin (1:10000 dilution; Sungenebiotech) antibody followed by horseradish peroxidase–conjugated secondary antibody, and detected by immunoblotting with the High-Sensitivity ECL Chemiluminescence Detection Kit (Vazyme) or Super ECL Detection Reagent (Yeasen Biotech).

### C-peptide and insulin content analysis

ePP-β cells and human islets were collected, counted by Countess II FL (Thermo Fisher Scientific), and lysed using radioimmunoprecipitation assay buffer (Beyotime). Supernatant samples containing total insulin and C-peptide were respectively detected by a human insulin immunoassay kit (EZassay, catalog no. HM200) and human C-peptide ultrasensitive ELISA kit (Mercodia, catalog no. 10-1141-01). The insulin and C-peptide content was normalized by the cell number.

### Glucose-stimulated insulin secretion

GSIS was performed according to a previously reported protocol (*6*). ePP-β cells and human islets were washed twice with 1 ml of KRBH buffer (128 mM NaCl, 5 mM KCl, 2.7 mM CaCl_2_, 1.2 mM MgCl_2_, 1 mM sodium phosphate buffer, 1.2 mM KH_2_PO_4_, 5 mM NaHCO_3_, 10 mM Hepes, and 0.1% BSA). Then, clusters were preincubated in 1 ml of KRBH buffer for 30 min to remove residual insulin. During incubation, all tube lids were left open for air exchange. Clusters were incubated in 300 μl of KRBH buffer with 2 mM glucose (low-glucose KRBH) for 1 hour. After incubation, 100 μl of the supernatant was collected for ELISA analysis (low-glucose sample). Then, clusters were incubated in KRBH with 16.8 mM glucose (high-glucose KRBH) for 1 hour, and 100 μl of supernatant was collected after incubation (high-glucose sample). Last, clusters were dispersed into single cells using Accutase for cell counting.

### Electron microscopic analysis

ePP-β clusters were fixed, standard processed, and analyzed by Life Sciences Institute Microscope Core. Then, the electron data for insulin granules and mitochondria were collected using a transmission electron microscope (HITACHI, HT7700).

### mtDNA analysis

To measure mtDNA content, DNA was extracted from hPSCs, ePPs, ePP-β cells, and human islets using the Quick-DNA MiniPrep Kit (ZYMO). The ratio of mitochondrial mtDNA 16*S* ribosomal RNA (rRNA) gene versus nuclear β*-2-microglobulin* (β*2M*) gene was determined by qPCR (*9*). Human-specific primers used for mtDNA 16*S* rRNA and β*2M* are shown in table S2.

### Karyotyping analysis

Karyotyping analysis by G-banding of ePPs at passages 12 and 24 was carried out by the Department of Prenatal Diagnosis (Screening) Center of Hangzhou Women’s Hospital (Hangzhou Maternity and Child Health Care Hospital). Detailed methods are as reported elsewhere.

### Mouse studies

Immunodeficient severe combined immunodeficient (SCID) beige mice were purchased from Beijing Vital River Laboratory Animal Technology. Male mice, aged 8 to 10 weeks, were used for transplantation and fed in the Animal Core, Zhejiang University, Hangzhou. Diabetic mice were rendered by administering STZ (45 mg kg^−1^) for five consecutive days via intraperitoneal injection. Mice were anaesthetized with isoflurane (RWD). About 5 million ePP-β cells were transplanted under the kidney capsule. For in vivo glucose challenge experiments, mice with grafts were fasted for 16 hours. Then, the serum before and 60 min following an intraperitoneal injection of d-glucose solution (3 g kg^−1^) was collected for ELISA analysis. For intraperitoneal glucose tolerance test (IPGTT), mice with or without grafts were fasted for 16 hours, and we recorded the fasted blood glucose level. After that, mice were injected with d-glucose solution (2 g kg^−1^). In addition, the mouse blood glucose level was recorded at the chosen time points after intraperitoneal injection. For immunofluorescent staining, kidneys with grafts were removed for cryosection 1 week after transplantation. All of the mouse studies were compliant with relevant ethical regulations about animal research.

### RNA-seq and data analysis

Total RNA was isolated from indicated samples using the Quick-RNA MiniPrep Kit (ZYMO). cDNA library construction and high-throughput sequencing were performed by Novogene, and paired-end sequencing was carried out with the Illumina HiSeq 2500. Clean reads were aligned to the human genome (GRCh38, hg38) using HISAT2 (v2.1.0) with the default parameter settings. Transcript assembly was performed by stringtie (v2.0), and expression of transcripts sharing each gene_id was quantified as transcripts per million (TPM). The differential expression analysis was performed by R package DESeq2 (*53*) from the Bioconductor project (http://bioconductor.org/). The GO and KEGG enrichment analyses were performed with R package clusterProfiler (*54*) from the Bioconductor project. Heatmaps were generated by R package pheatmap from CRAN (https://cran.r-project.org/).

### CUT&Tag assay

CUT&Tag assay was performed as follows. First, ConA Beads (CST, #93569) were pipetted into a new tube (10 μl per sample) and washed with activation buffer. Cells (1 × 10^5^) on day 7 were harvested and washed with wash buffer. The cells were mixed with ConA Beads and incubated with anti-Brd4 antibody (1:100; Abcam) overnight at 4°C. Donkey anti-rabbit immunoglobulin G antibody (Invitrogen) was diluted at 1:100 and added into the samples. Then, the samples were incubated for 1 hour at room temperature. After washing away the unbounded secondary antibody, Hyperactive pG-Tn5 Transposase (Vazyme) was added and incubated with cells for 1 hour at room temperature. The samples were washed with Dig-med buffer and followed with tagmentation by tagmentation buffer. Next, DNA was isolated by phenol-chloroform and dissolved in TE buffer. DNA was amplified with N5 and N7 primers and purified with VAHTS DNA Clean Beads (Vazyme) for high-throughput sequencing.

### CUT&Tag analysis

Paired-end sequencing was carried out with the Illumina NovaSeq 6000 with read length of 150 base pairs (bp). Clean reads were aligned to the human genome (GRCh38, hg38) using Bowtie2 (v2.2.5) with the following options: --local --very-sensitive-local --no-unal --no-mixed –nodiscordant --phred33 -I 10 -X 700. For peak calling, parameters used were macs2 (v2.1.2) callpeak -p 1e-5 -f BAMPE -g hs -n sample-name -B --keep-dup all. The peak visualization in genome was shown by Integrative Genomics Viewer (IGV) software (*55*). In addition, the peak annotations and heatmaps were performed with R package ChIPseeker (*56*) from the Bioconductor project. Motif enrichment was done using HOMER (v4.11) with the following options: -fdr 0.05 hg38 -len 8,10,12. Bigwig files were generated with the bamCoverage function in deepTools (v3.4.3) with default parameter settings.

### Assay for transposase-accessible chromatin using sequencing

Cells (5 × 10^4^) were collected and lysed using lysis buffer [10 mM tris-HCl (pH 7.4), 10 mM NaCl, 3 mM MgCl_2_, and 0.1% NP-40] to collect cell nuclei. Then, the standard processing was performed using the TruePrep DNA Library Prep Kit V2 for Illumina (Vazyme). The library concentration was measured using the Qubit Double-Stranded DNA BR Assay Kit (Invitrogen) by Qubit (Invitrogen).

### ATAC-seq analysis

High-throughput sequencing was performed by Novogene, and paired-end sequencing was carried out with the Illumina Novaseq 6000 with read length of 200 bp. Clean reads were aligned to the human genome (GRCh38, hg38) using Bowtie2 (v2.2.5) with the following options: --no-discordant --no-unal --no-mixed -X 2000 --local. For removing the null and not unmapped reads, the sambamba (v0.7.1) was performed with default parameter settings. The MarkDuplicates function in picard-tools (v2.20.5) was used for removing the duplicated reads. Mitochondrial reads were removed by samtools (v1.9). For peak calling, parameters used were macs2 (v2.1.2) callpeak: -p 1e-5 --nomodel --shift -100 --extsize 200. The peak visualization in genome was shown by IGV software (*55*). In addition, the peak annotations and heatmaps were performed with R package ChIPseeker (*56*) from the Bioconductor project. Motif enrichment was done using HOMER (v4.11) with the following options: -fdr 0.05 hg38 -len 8,10,12. Bigwig files were generated with the bamCoverage function in deepTools (v3.4.3) with default parameter settings.

### Chromatin immunoprecipitation combined with sequencing

Cells cultured in a 10-cm dish (about 10^7^ cells) were collected and fixed in 1% formaldehyde at room temperature for 10 min. Then, 0.125 M glycine was used to quench the reaction. After washing with PBS twice, cells were harvested and lysed by lysis buffer and cell lysis buffer in the Magna ChIP A/G One-Day Chromatin Immunoprecipitation Kit (EMD Millipore Corp., catalog no. 17-10086) step by step. Thereafter, the nuclear pellets were resuspended in nuclear lysis buffer in the Magna ChIP A/G One-Day Chromatin Immunoprecipitation Kit. The mixture was sonicated as 30 s on and 30 s off with 20 cycles. The sonication samples were centrifuged at 12,000 rpm for 20 min at 4°C to harvest supernatant. Protein A/G magnetic beads (20 μl; EMD Millipore Corp) for each immunoprecipitation were washed with block buffer (1× PBST and 0.5% BSA) twice. Before immunoprecipitation, protein A/G magnetic beads and 2 μg of antibodies were incubated at 4°C overnight followed by washing using block solution three times. Moreover, the sonication supernatant was diluted in dilution buffer to 1-ml total volume, and 20 μl of the mixture was left as input samples. Next, the sonication supernatant was incubated with beads at 4°C for 6 to 10 hours. After incubation, the protein A/G beads, antibody, and chromatin formed the complex. In addition, the complex was stepwise washed in 1 ml of wash buffer [20 mM tris-HCl (pH 8), 150 mM NaCl, 2 mM EDTA, 0.1% SDS, and 1% Triton X-100], high-NaCl buffer (wash supplemented with 500 mM NaCl), and TE buffer [10 mM tris-HCl (pH 8), 50 mM NaCl, and 1 mM EDTA] at 4°C for 5 min. Then, chromatin was eluted by incubating with 200 μl of elution buffer [50 mM tris-HCl (pH 8), 10 mM EDTA, and 1% SDS] at 65°C for 30 min followed by reversing in elution buffer with 0.2 M NaCl at 65°C overnight. Last, RNA in the samples was digested with ribonuclease A (0.2 mg ml^−1^) at 37°C for 2 hours, and proteins were digested with proteinase K (0.2 mg ml^−1^) at 62°C for 2 hours and 95°C for 10 min. DNA was extracted by using the DNA Clean & Concentrator-5 Kit (ZYMO, catalog no. D4014). ChIP and input DNA were quantified by Qubit before library preparation using the VAHTS Universal DNA Library Prep Kit for Illumina V2 (Vazyme).

### ChIP-seq analysis

High-throughput sequencing was performed by Novogene, and paired-end sequencing was carried out with the Illumina Novaseq 6000 with read length of 200 bp. Clean reads were aligned to the human genome (GRCh38, hg38) using Bowtie2 (v2.2.5) with options -3 5 --local. For removing the null and not unmapped reads, the sambamba (v0.7.1) was performed with default parameter settings. The MarkDuplicates function in picard-tools (v2.20.5) was used for removing the duplicated reads. Mitochondrial reads were removed by samtools (v1.9). For peak calling, macs2 (v2.1.2) was also used with default parameter settings. The peak visualization in genome was shown by IGV software (*55*). In addition, the peak annotations and heatmaps were performed with R package ChIPseeker (*56*) from the Bioconductor project. Motif enrichment was done using HOMER (v4.11) with the following options: -fdr 0.05 hg38 -len 8,10,12. Bigwig files were generated with the bamCoverage function in deepTools (v3.4.3) with default parameter settings.

### Statistics and reproducibility

The specific statistical analysis is described in the corresponding figure legends. Two-tailed unpaired *t* tests (Student’s *t* test) were used to obtain the *P* values. Data analysis was performed in GraphPad Prism version 7. The following convention was used for indicating *P* values: not significant (ns), *P* > 0.05; *P < 0.05; **P < 0.01; ***P < 0.001; and ****P < 0.0001. All data are shown as the means with error bars representing the SD.

In this manuscript, representative microscopy images are shown, but these were replicated and confirmed by several independent experiments as follows. In Fig. 1D: This staining result was replicated in four independent differentiations. In Fig. 2D: This karyotype image was repeated in two independent experiments; Fig. 2E: this staining result was replicated in three independent experiments; Fig. 2H: staining results were obtained from two independent immunostaining. In Fig. 3B: Staining results were repeated in three independent differentiations; Fig. 3, H and K: these transmission electron micrographs were reproduced in two independent experiments; Fig. 3J: this staining result was replicated in three independent experiments. In Fig. 4F: This staining result was replicated in three independent experiments. In fig. S1A: This differentiation method was repeated numerous times (>30) as it was the basis of all the experiments in Fig. 1 and figs. S1 and S2; fig. S1C: this staining result was reproduced by more than five independent experiments. In fig. S2B: This staining result was reproduced by three independent experiments. In fig. S3B: These bright-field images were reproduced in three independent experiments; fig. S3D: this staining result was repeated three times; fig. S3F: these bright-field images were repeated in three independent experiments; fig. S3G: this Western blotting result represented three independent experiments; fig. S3I: this staining result was reproduced three times. In fig. S4A: These bright-field images were reproduced in three independent experiments; fig. S4B: this staining result was replicated in three independent differentiations; fig. S4D: this karyotype image was repeated in two independent experiments; fig. S4, E and F: these staining results were repeated in three independent differentiations. In fig. S5, A and E: These staining results were reproduced in three independent experiments. In fig. S6, A and E: These staining results were reproduced in three independent experiments.

## Notes

View/request a protocol for this paper from *Bio-protocol*.

## References

[1] A. M. J. Shapiro, C. Ricordi, B. J. Hering, H. Auchincloss, R. Lindblad, R. P. Robertson, A. Secchi, M. D. Brendel, T. Berney, D. C. Brennan, E. Cagliero, R. Alejandro, E. A. Ryan, B. D. Mercurio, P. Morel, K. S. Polonsky, J.-A. Reems, R. G. Bretzel, F. Bertuzzi, T. Froud, R. Kandaswamy, D. E. R. Sutherland, G. Eisenbarth, M. Segal, J. Preiksaitis, G. S. Korbutt, F. B. Barton, L. Viviano, V. Seyfert-Margolis, J. Bluestone, J. R. T. Lakey, International trial of the edmonton protocol for islet transplantation. N. Engl. J. Med. 355, 1318–1330 (2006). 17005949 10.1056/NEJMoa061267

[2] C. E. Murry, G. Keller, Differentiation of embryonic stem cells to clinically relevant populations: Lessons from embryonic development. Cell 132, 661–680 (2008). 18295582 10.1016/j.cell.2008.02.008

[3] Z. Zhou, X. Ma, S. Zhu, Recent advances and potential applications of human pluripotent stem cell-derived pancreatic β cells. Acta Biochim Biophys Sin 52, 708–715 (2020). 32445468 10.1093/abbs/gmaa047

[4] E. Kroon, L. A. Martinson, K. Kadoya, A. G. Bang, O. G. Kelly, S. Eliazer, H. Young, M. Richardson, N. G. Smart, J. Cunningham, A. D. Agulnick, K. A. D’Amour, M. K. Carpenter, E. E. Baetge, Pancreatic endoderm derived from human embryonic stem cells generates glucose-responsive insulin-secreting cells in vivo. Nat. Biotechnol. 26, 443–452 (2008). 18288110 10.1038/nbt1393

[5] A. Rezania, J. E. Bruin, P. Arora, A. Rubin, I. Batushansky, A. Asadi, S. O’Dwyer, N. Quiskamp, M. Mojibian, T. Albrecht, Y. H. Yang, J. D. Johnson, T. J. Kieffer, Reversal of diabetes with insulin-producing cells derived in vitro from human pluripotent stem cells. Nat. Biotechnol. 32, 1121–1133 (2014). 25211370 10.1038/nbt.3033

[6] F. W. Pagliuca, J. R. Millman, M. Gurtler, M. Segel, A. Van Dervort, J. H. Ryu, Q. P. Peterson, D. Greiner, D. A. Melton, Generation of functional human pancreatic β cells in vitro. Cell 159, 428–439 (2014). 25303535 10.1016/j.cell.2014.09.040 PMC4617632

[7] H. A. Russ, A. V. Parent, J. J. Ringler, T. G. Hennings, G. G. Nair, M. Shveygert, T. Guo, S. Puri, L. Haataja, V. Cirulli, R. Blelloch, G. L. Szot, P. Arvan, M. Hebrok, Controlled induction of human pancreatic progenitors produces functional beta-like cells in vitro. EMBO J. 34, 1759–1772 (2015). 25908839 10.15252/embj.201591058 PMC4516429

[8] M. C. Nostro, F. Sarangi, C. Yang, A. Holland, A. G. Elefanty, E. G. Stanley, D. L. Greiner, G. Keller, Efficient generation of NKX6-1+ pancreatic progenitors from multiple human pluripotent stem cell lines. Stem Cell Rep. 4, 591–604 (2015). 10.1016/j.stemcr.2015.02.017 PMC4400642 25843049

[9] G. G. Nair, J. S. Liu, H. A. Russ, S. Tran, M. S. Saxton, R. Chen, C. Juang, M. L. Li, V. Q. Nguyen, S. Giacometti, S. Puri, Y. Xing, Y. Wang, G. L. Szot, J. Oberholzer, A. Bhushan, M. Hebrok, Recapitulating endocrine cell clustering in culture promotes maturation of human stem-cell-derived β cells. Nat. Cell Biol. 21, 263–274 (2019). 30710150 10.1038/s41556-018-0271-4 PMC6746427

[10] J. R. Alvarez-Dominguez, J. Donaghey, N. Rasouli, J. H. R. Kenty, A. Helman, J. Charlton, J. R. Straubhaar, A. Meissner, D. A. Melton, Circadian entrainment triggers maturation of human in vitro islets. Cell Stem Cell 26, 108–122.e10 (2020). 31839570 10.1016/j.stem.2019.11.011

[11] N. J. Hogrebe, P. Augsornworawat, K. G. Maxwell, L. Velazco-Cruz, J. R. Millman, Targeting the cytoskeleton to direct pancreatic differentiation of human pluripotent stem cells. Nat. Biotechnol. 38, 460–470 (2020). 32094658 10.1038/s41587-020-0430-6 PMC7274216

[12] P. U. Mahaddalkar, K. Scheibner, S. Pfluger, Ansarullah, M. Sterr, J. Beckenbauer, M. Irmler, J. Beckers, S. Knobel, H. Lickert, Generation of pancreatic β cells from CD177^+^ anterior definitive endoderm. Nat. Biotechnol. 38, 1061–1072 (2020). 32341565 10.1038/s41587-020-0492-5

[13] A. Mamidi, C. Prawiro, P. A. Seymour, K. H. de Lichtenberg, A. Jackson, P. Serup, H. Semb, Mechanosignalling via integrins directs fate decisions of pancreatic progenitors. Nature 564, 114–118 (2018). 30487608 10.1038/s41586-018-0762-2

[14] K. A. D’Amour, A. G. Bang, S. Eliazer, O. G. Kelly, A. D. Agulnick, N. G. Smart, M. A. Moorman, E. Kroon, M. K. Carpenter, E. E. Baetge, Production of pancreatic hormone-expressing endocrine cells from human embryonic stem cells. Nat. Biotechnol. 24, 1392–1401 (2006). 17053790 10.1038/nbt1259

[15] D. Zhang, W. Jiang, M. Liu, X. Sui, X. Yin, S. Chen, Y. Shi, H. Deng, Highly efficient differentiation of human ES cells and iPS cells into mature pancreatic insulin-producing cells. Cell Res. 19, 429–438 (2009). 19255591 10.1038/cr.2009.28

[16] E. Yoshihara, C. O’Connor, E. Gasser, Z. Wei, T. G. Oh, T. W. Tseng, D. Wang, F. Cayabyab, Y. Dai, R. T. Yu, C. Liddle, A. R. Atkins, M. Downes, R. M. Evans, Immune-evasive human islet-like organoids ameliorate diabetes. Nature 586, 606–611 (2020). 32814902 10.1038/s41586-020-2631-z PMC7872080

[17] L. Velazco-Cruz, J. Song, K. G. Maxwell, M. M. Goedegebuure, P. Augsornworawat, N. J. Hogrebe, J. R. Millman, Acquisition of dynamic function in human stem cell-derived β cells. Stem Cell Rep. 12, 351–365 (2019). 10.1016/j.stemcr.2018.12.012 PMC6372986 30661993

[18] E. Conrad, R. Stein, C. S. Hunter, Revealing transcription factors during human pancreatic β cell development. Trends Endocrinol. Metab. 25, 407–414 (2014). 24831984 10.1016/j.tem.2014.03.013 PMC4167784

[19] G. Nair, M. Hebrok, Islet formation in mice and men: Lessons for the generation of functional insulin-producing β-cells from human pluripotent stem cells. Curr. Opin. Genet. Dev. 32, 171–180 (2015). 25909383 10.1016/j.gde.2015.03.004 PMC4523641

[20] M. F. Offield, T. L. Jetton, P. A. Labosky, M. Ray, R. W. Stein, M. A. Magnuson, B. L. Hogan, C. V. Wright, PDX-1 is required for pancreatic outgrowth and differentiation of the rostral duodenum. Development 122, 983–995 (1996). 8631275 10.1242/dev.122.3.983

[21] J. Jonsson, L. Carlsson, T. Edlund, H. Edlund, Insulin-promoter-factor 1 is required for pancreas development in mice. Nature 371, 606–609 (1994). 7935793 10.1038/371606a0

[22] A. E. Schaffer, K. K. Freude, S. B. Nelson, M. Sander, Nkx6 transcription factors and Ptf1a function as antagonistic lineage determinants in multipotent pancreatic progenitors. Dev. Cell 18, 1022–1029 (2010). 20627083 10.1016/j.devcel.2010.05.015 PMC3133668

[23] R. E. Jennings, A. A. Berry, R. Kirkwood-Wilson, N. A. Roberts, T. Hearn, R. J. Salisbury, J. Blaylock, K. Piper Hanley, N. A. Hanley, Development of the human pancreas from foregut to endocrine commitment. Diabetes 62, 3514–3522 (2013). 23630303 10.2337/db12-1479 PMC3781486

[24] X. Cheng, L. Ying, L. Lu, A. M. Galvao, J. A. Mills, H. C. Lin, D. N. Kotton, S. S. Shen, M. C. Nostro, J. K. Choi, M. J. Weiss, D. L. French, P. Gadue, Self-renewing endodermal progenitor lines generated from human pluripotent stem cells. Cell Stem Cell 10, 371–384 (2012). 22482503 10.1016/j.stem.2012.02.024 PMC3580854

[25] J. B. Sneddon, M. Borowiak, D. A. Melton, Self-renewal of embryonic-stem-cell-derived progenitors by organ-matched mesenchyme. Nature 491, 765–768 (2012). 23041930 10.1038/nature11463 PMC6005657

[26] N. R. Hannan, R. P. Fordham, Y. A. Syed, V. Moignard, A. Berry, R. Bautista, N. A. Hanley, K. B. Jensen, L. Vallier, Generation of multipotent foregut stem cells from human pluripotent stem cells. Stem Cell Rep. 1, 293–306 (2013). 10.1016/j.stemcr.2013.09.003 PMC3849417 24319665

[27] J. Trott, E. K. Tan, S. Ong, D. M. Titmarsh, S. Denil, M. Giam, C. K. Wong, J. Wang, M. Shboul, M. Eio, J. Cooper-White, S. M. Cool, G. Rancati, L. W. Stanton, B. Reversade, N. R. Dunn, Long-term culture of self-renewing pancreatic progenitors derived from human pluripotent stem cells. Stem Cell Rep. 8, 1675–1688 (2017). 10.1016/j.stemcr.2017.05.019 PMC5470345 28591650

[28] M. Bakhti, K. Scheibner, S. Tritschler, A. Bastidas-Ponce, M. Tarquis-Medina, F. J. Theis, H. Lickert, Establishment of a high-resolution 3D modeling system for studying pancreatic epithelial cell biology in vitro. Mol. Metab. 30, 16–29 (2019). 31767167 10.1016/j.molmet.2019.09.005 PMC6812400

[29] C. A. Goncalves, M. Larsen, S. Jung, J. Stratmann, A. Nakamura, M. Leuschner, L. Hersemann, R. Keshara, S. Perlman, L. Lundvall, L. L. Thuesen, K. J. Hare, I. Amit, A. Jorgensen, Y. H. Kim, A. Del Sol, A. Grapin-Botton, A 3D system to model human pancreas development and its reference single-cell transcriptome atlas identify signaling pathways required for progenitor expansion. Nat. Commun. 12, 3144 (2021). 34035279 10.1038/s41467-021-23295-6 PMC8149728

[30] L. Huang, B. Bockorny, I. Paul, D. Akshinthala, P. O. Frappart, O. Gandarilla, A. Bose, V. Sanchez-Gonzalez, E. E. Rouse, S. D. Lehoux, N. Pandell, C. M. Lim, J. G. Clohessy, J. Grossman, R. Gonzalez, S. P. Del Pino, G. Daaboul, M. S. Sawhney, S. D. Freedman, A. Kleger, R. D. Cummings, A. Emili, L. B. Muthuswamy, M. Hidalgo, S. K. Muthuswamy, PDX-derived organoids model in vivo drug response and secrete biomarkers. JCI Insight 5, e135544 (2020). 32990680 10.1172/jci.insight.135544 PMC7710298

[31] J. Ameri, R. Borup, C. Prawiro, C. Ramond, K. A. Schachter, R. Scharfmann, H. Semb, Efficient generation of glucose-responsive beta cells from isolated GP2(+) human pancreatic progenitors. Cell Rep. 19, 36–49 (2017). 28380361 10.1016/j.celrep.2017.03.032

[32] S. Zhu, H. A. Russ, X. Wang, M. Zhang, T. Ma, T. Xu, S. Tang, M. Hebrok, S. Ding, Human pancreatic beta-like cells converted from fibroblasts. Nat. Commun. 7, 10080 (2016). 26733021 10.1038/ncomms10080 PMC4729817

[33] W. Li, K. Li, W. Wei, S. Ding, Chemical approaches to stem cell biology and therapeutics. Cell Stem Cell 13, 270–283 (2013). 24012368 10.1016/j.stem.2013.08.002 PMC3898630

[34] X. Ma, L. Kong, S. Zhu, Reprogramming cell fates by small molecules. Protein Cell 8, 328–348 (2017). 28213718 10.1007/s13238-016-0362-6 PMC5413596

[35] S. Chen, M. Borowiak, J. L. Fox, R. Maehr, K. Osafune, L. Davidow, K. Lam, L. F. Peng, S. L. Schreiber, L. L. Rubin, D. Melton, A small molecule that directs differentiation of human ESCs into the pancreatic lineage. Nat. Chem. Biol. 5, 258–265 (2009). 19287398 10.1038/nchembio.154

[36] D. Sakano, N. Shiraki, K. Kikawa, T. Yamazoe, M. Kataoka, K. Umeda, K. Araki, D. Mao, S. Matsumoto, N. Nakagata, O. Andersson, D. Stainier, F. Endo, K. Kume, M. Uesugi, S. Kume, VMAT2 identified as a regulator of late-stage β-cell differentiation. Nat. Chem. Biol. 10, 141–148 (2014). 24316738 10.1038/nchembio.1410

[37] Z. Ghazizadeh, D. I. Kao, S. Amin, B. Cook, S. Rao, T. Zhou, T. Zhang, Z. Xiang, R. Kenyon, O. Kaymakcalan, C. Liu, T. Evans, S. Chen, ROCKII inhibition promotes the maturation of human pancreatic beta-like cells. Nat. Commun. 8, 298 (2017). 28824164 10.1038/s41467-017-00129-y PMC5563509

[38] X. Ma, S. Zhu, Chemical strategies for pancreatic β cell differentiation, reprogramming, and regeneration. Acta Biochim Biophys Sin 49, 289–301 (2017). 28338772 10.1093/abbs/gmx008

[39] S. Zhu, W. Wei, S. Ding, Chemical strategies for stem cell biology and regenerative medicine. Annu. Rev. Biomed. Eng. 13, 73–90 (2011). 21513460 10.1146/annurev-bioeng-071910-124715

[40] K. Liu, C. Yu, M. Xie, K. Li, S. Ding, Chemical modulation of cell fate in stem cell therapeutics and regenerative medicine. Cell Chem. Biol. 23, 893–916 (2016). 27524294 10.1016/j.chembiol.2016.07.007

[41] M. A. Dawson, R. K. Prinjha, A. Dittmann, G. Giotopoulos, M. Bantscheff, W. I. Chan, S. C. Robson, C. W. Chung, C. Hopf, M. M. Savitski, C. Huthmacher, E. Gudgin, D. Lugo, S. Beinke, T. D. Chapman, E. J. Roberts, P. E. Soden, K. R. Auger, O. Mirguet, K. Doehner, R. Delwel, A. K. Burnett, P. Jeffrey, G. Drewes, K. Lee, B. J. Huntly, T. Kouzarides, Inhibition of BET recruitment to chromatin as an effective treatment for MLL-fusion leukaemia. Nature 478, 529–533 (2011). 21964340 10.1038/nature10509 PMC3679520

[42] P. Filippakopoulos, J. Qi, S. Picaud, Y. Shen, W. B. Smith, O. Fedorov, E. M. Morse, T. Keates, T. T. Hickman, I. Felletar, M. Philpott, S. Munro, M. R. McKeown, Y. Wang, A. L. Christie, N. West, M. J. Cameron, B. Schwartz, T. D. Heightman, N. La Thangue, C. A. French, O. Wiest, A. L. Kung, S. Knapp, J. E. Bradner, Selective inhibition of BET bromodomains. Nature 468, 1067–1073 (2010). 20871596 10.1038/nature09504 PMC3010259

[43] V. K. Wimalasena, T. Wang, L. H. Sigua, A. D. Durbin, J. Qi, Using chemical epigenetics to target cancer. Mol. Cell 78, 1086–1095 (2020). 32407673 10.1016/j.molcel.2020.04.023 PMC8033568

[44] T. Guo, L. Landsman, N. Li, M. Hebrok, Factors expressed by murine embryonic pancreatic mesenchyme enhance generation of insulin-producing cells from hESCs. Diabetes 62, 1581–1592 (2013). 23305648 10.2337/db12-0167 PMC3636645

[45] R. Di Micco, B. Fontanals-Cirera, V. Low, P. Ntziachristos, S. K. Yuen, C. D. Lovell, I. Dolgalev, Y. Yonekubo, G. Zhang, E. Rusinova, G. Gerona-Navarro, M. Canamero, M. Ohlmeyer, I. Aifantis, M. M. Zhou, A. Tsirigos, E. Hernando, Control of embryonic stem cell identity by BRD4-dependent transcriptional elongation of super-enhancer-associated pluripotency genes. Cell Rep. 9, 234–247 (2014). 25263550 10.1016/j.celrep.2014.08.055 PMC4317728

[46] J. I. Sakamaki, S. Wilkinson, M. Hahn, N. Tasdemir, J. O’Prey, W. Clark, A. Hedley, C. Nixon, J. S. Long, M. New, T. Van Acker, S. A. Tooze, S. W. Lowe, I. Dikic, K. M. Ryan, Bromodomain protein BRD4 is a transcriptional repressor of autophagy and lysosomal function. Mol. Cell 66, 517–532.e9 (2017). 28525743 10.1016/j.molcel.2017.04.027 PMC5446411

[47] R. J. Conrad, P. Fozouni, S. Thomas, H. Sy, Q. Zhang, M. M. Zhou, M. Ott, The short isoform of BRD4 promotes HIV-1 latency by engaging repressive SWI/SNF chromatin-remodeling complexes. Mol. Cell 67, 1001–1012.e6 (2017). 28844864 10.1016/j.molcel.2017.07.025 PMC5610089

[48] L. W. S. Finley, S. A. Vardhana, B. W. Carey, D. Alonso-Curbelo, R. Koche, Y. Chen, D. Wen, B. King, M. R. Radler, S. Rafii, S. W. Lowe, C. D. Allis, C. B. Thompson, Pluripotency transcription factors and Tet1/2 maintain Brd4-independent stem cell identity. Nat. Cell Biol. 20, 565–574 (2018). 29662175 10.1038/s41556-018-0086-3 PMC5937285

[49] X. Ma, X. Chen, Y. Jin, W. Ge, W. Wang, L. Kong, J. Ji, X. Guo, J. Huang, X. H. Feng, J. Fu, S. Zhu, Small molecules promote CRISPR-Cpf1-mediated genome editing in human pluripotent stem cells. Nat. Commun. 9, 1303 (2018). 29610531 10.1038/s41467-018-03760-5 PMC5880812

[50] F. Gonzalez, Z. Zhu, Z. D. Shi, K. Lelli, N. Verma, Q. V. Li, D. Huangfu, An iCRISPR platform for rapid, multiplexable, and inducible genome editing in human pluripotent stem cells. Cell Stem Cell 15, 215–226 (2014). 24931489 10.1016/j.stem.2014.05.018 PMC4127112

[51] A. K. Teo, A. J. Wagers, R. N. Kulkarni, New opportunities: Harnessing induced pluripotency for discovery in diabetes and metabolism. Cell Metab. 18, 775–791 (2013). 24035588 10.1016/j.cmet.2013.08.010 PMC3858409

[52] S. Zhu, W. Li, H. Zhou, W. Wei, R. Ambasudhan, T. Lin, J. Kim, K. Zhang, S. Ding, Reprogramming of human primary somatic cells by OCT4 and chemical compounds. Cell Stem Cell 7, 651–655 (2010). 21112560 10.1016/j.stem.2010.11.015 PMC3812930

[53] M. I. Love, W. Huber, S. Anders, Moderated estimation of fold change and dispersion for RNA-seq data with DESeq2. Genome Biol. 15, 550 (2014). 25516281 10.1186/s13059-014-0550-8 PMC4302049

[54] G. Yu, L. G. Wang, Y. Han, Q. Y. He, clusterProfiler: An R package for comparing biological themes among gene clusters. OMICS 16, 284–287 (2012). 22455463 10.1089/omi.2011.0118 PMC3339379

[55] J. T. Robinson, H. Thorvaldsdottir, W. Winckler, M. Guttman, E. S. Lander, G. Getz, J. P. Mesirov, Integrative genomics viewer. Nat. Biotechnol. 29, 24–26 (2011). 21221095 10.1038/nbt.1754 PMC3346182

[56] G. Yu, L. G. Wang, Q. Y. He, ChIPseeker: An R/Bioconductor package for ChIP peak annotation, comparison and visualization. Bioinformatics 31, 2382–2383 (2015). 25765347 10.1093/bioinformatics/btv145

