# Supplementary material for: Human expandable pancreatic progenitor–derived β cells ameliorate diabetes
Source: Sci Adv. 2022 Feb 23;8(8):eabk1826. doi: 10.1126/sciadv.abk1826 (PMC8865776; doi:10.1126/sciadv.abk1826)
Supplement: Supplementary file 1 — Figs. S1 to S7 Tables S1 to S3 [file sciadv.abk1826_sm.pdf]

Science Advances
AAAS

Supplementary Materials for

Human expandable pancreatic progenitor–derived β cells ameliorate diabetes

Xiaojie Ma, Yunkun Lu, Ziyu Zhou, Qin Li, Xi Chen, Weiyun Wang, Yan Jin, Zhensheng Hu, Guo Chen, Qian Deng, Weina Shang, Hao Wang, Hongxing Fu, Xiangwei He, Xin-Hua Feng, Saiyong Zhu*

*Corresponding author. Email: saiyong@zju.edu.cn

Published 23 February 2022, *Sci. Adv.* **8**, eabk1826 (2022)
DOI: 10.1126/sciadv.abk1826

This PDF file includes:

Figs. S1 to S7
Tables S1 to S3

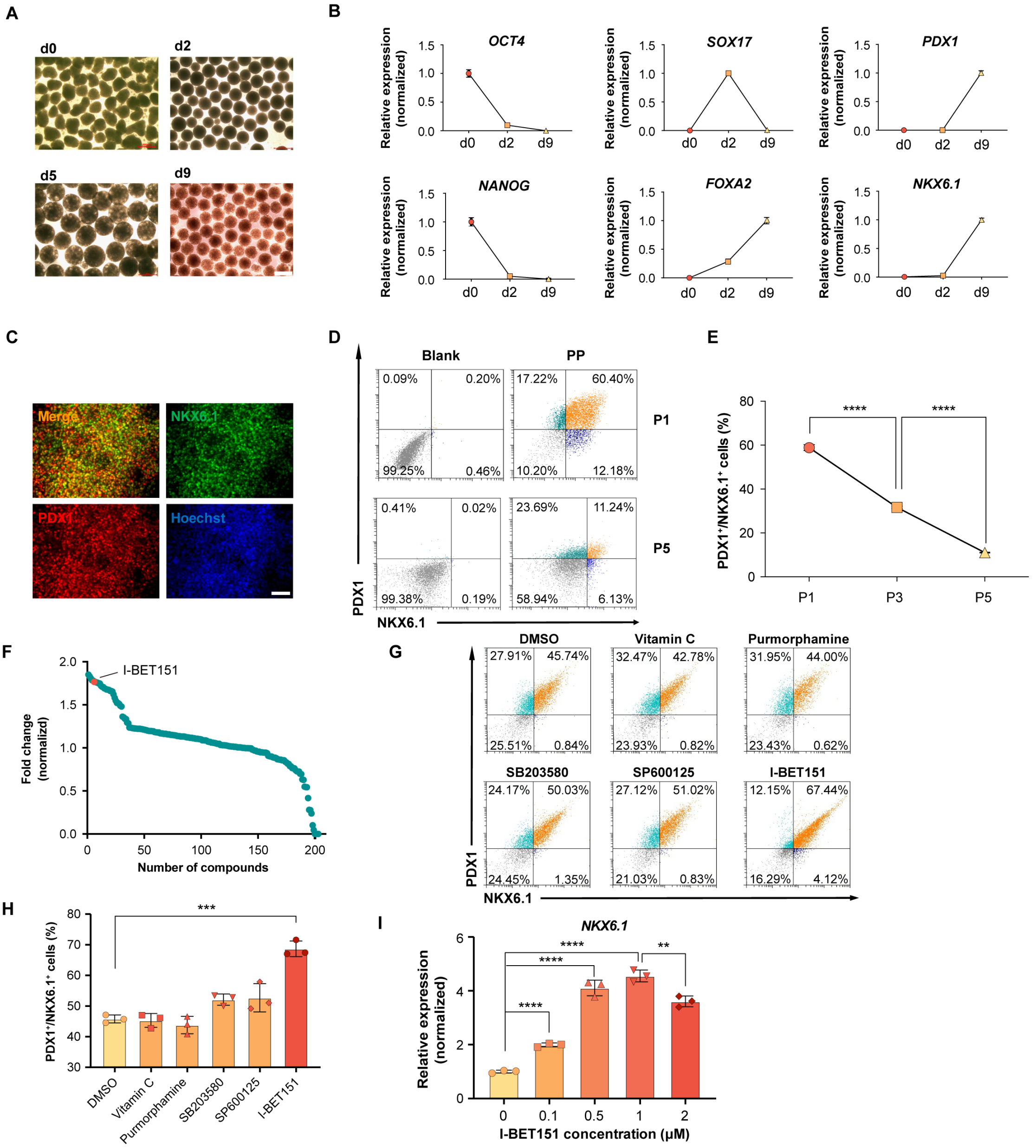

Fig. S1. Derivation of pancreatic progenitors from hPSCs. (**A**) Bright field of the pancreatic differentiation process. Scale bar, 500 μm. (**B**) RT-qPCR analysis of stage-specific marker gene expression. hPSC marker genes, *OCT4* and *NANOG*; DE marker genes, *SOX17* and *FOXA2*; PP marker genes, *PDX1* and *NKX6.1* (N = 3). (**C**) Immunofluorescent staining indicated that the majority of PPs differentiated from hPSCs were double-positive for PDX1 and NKX6.1 (N = 3). Scale bar, 200 μm. (**D**) Representative flow cytometry of PPs cultured in basal medium (EF6) at passage 1 (P1) and passage 5 (P5). The percentage of PDX1$^+$NKX6.1$^+$ PPs decreased rapidly during passaging (N = 3). (**E**) The percentage of PDX1$^+$NKX6.1$^+$ cells in the cell

populations at P1, P3, and P5. P1, passage 1; P3, passage 3; P5, passage 5 (*N* = 3). (**F**) Results of the first-round chemical screening. 203 small molecules were screened and I-BET151, which was shown in red, was one of the top candidates. (**G** and **H**) FACS demonstrated that I-BET151 significantly increased the percentage of $PDX1^+NKX6.1^+$ cells (*N* = 3). (**I**) RT-qPCR analysis of *NKX6.1* gene expression in PPs treated with different concentration of I-BET151 (*N* = 3). All data are expressed as mean ± s.d. Statistical significance calculated using two-tailed Student's *t*-test, ** $p < 0.01$, *** $p < 0.001$, **** $p < 0.0001$.

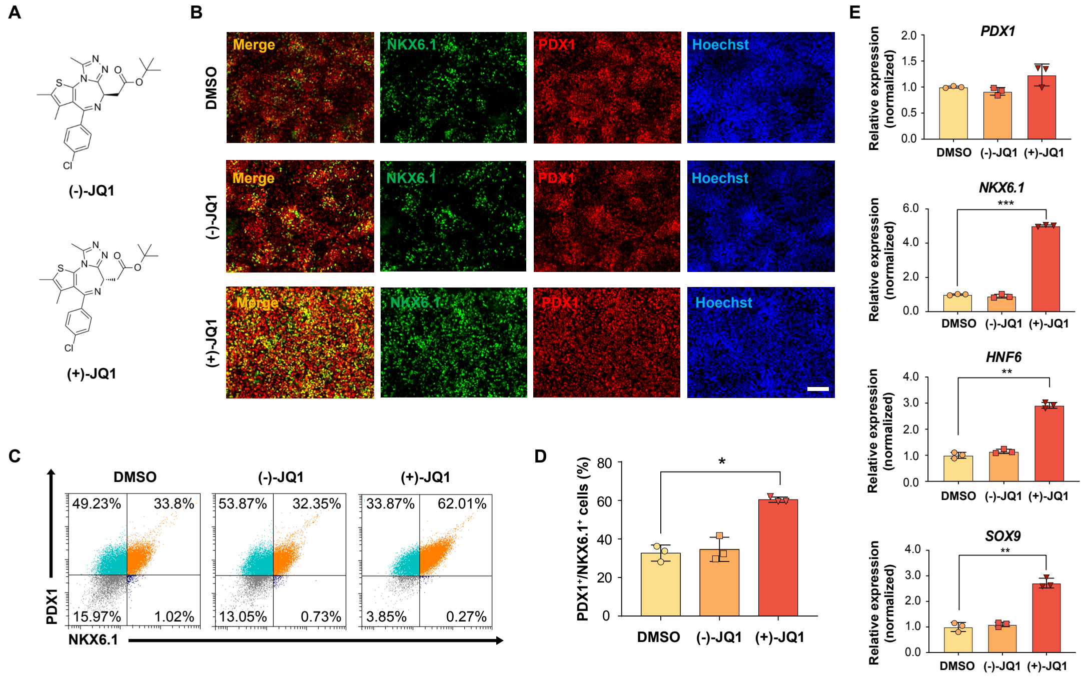

Fig. S2. Another BET inhibitor (+)-JQ1 also promoted human pancreatic progenitor expansion. (**A**) Chemical structure of another BET inhibitor (+)-JQ1 and inactive stereoisomer (-)-JQ1. (**B**) Representative immunofluorescent staining of PPs for PDX1, NKX6.1, and nuclei demonstrated that 1 μM (+)-JQ1 could increase the percentage of PDX1$^+$NKX6.1$^+$ cells but 1 μM (-)-JQ1 could not. The basal medium was EF6. Scale bar, 200 μm. (**C** and **D**) Representative flow cytometry dot plots (C) and population percentages (D) of PDX1$^+$NKX6.1$^+$ cells treated with (-)-JQ1 and (+)-JQ1, respectively (N = 3). (**E**) RT-qPCR analysis of *PDX1*, *NKX6.1*, *HNF6*, and *SOX9* gene expression in PPs treated with (-)-JQ1 and (+)-JQ1, respectively (N = 3). All data are expressed as mean ± s.d. Statistical significance calculated using two-tailed Student's *t*-test, * $p < 0.05$, ** $p < 0.01$, *** $p < 0.001$.

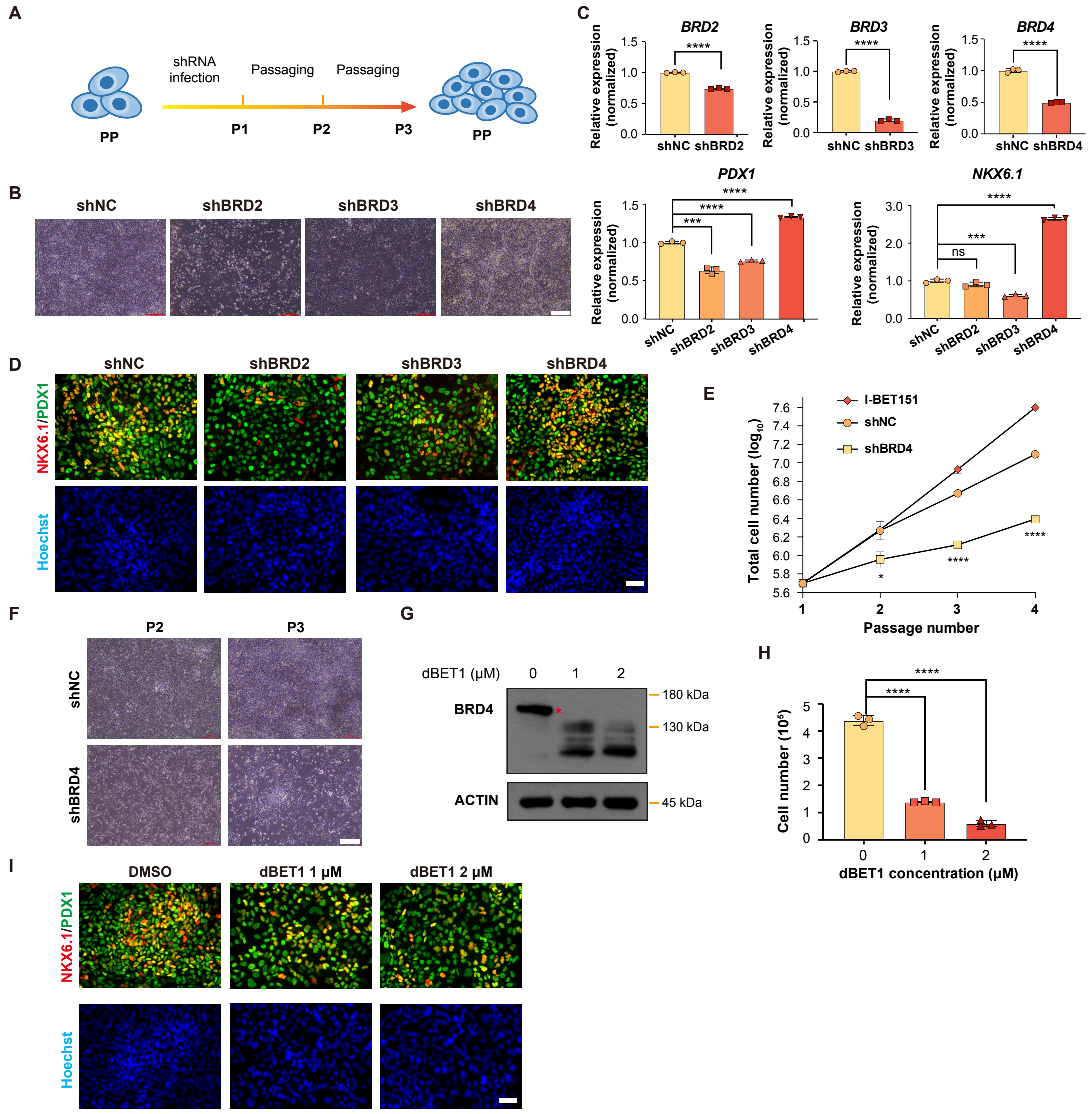

Fig. S3. BRD4 protein is essential for PP maintenance. (**A**) Schematic of knocking down *BRD2*, *BRD3*, and *BRD4* in PPs by shRNAs. (**B**) Bright-field images of PPs in shNC, shBRD2, shBRD3, and shBRD4 groups at passage 1, respectively. Scale bar, 500 μm. (**C**) RT-qPCR analysis for the expression of *BRD2*, *BRD3*, *BRD4*, *PDX1*, and *NKX6.1* after knockdown by shRNAs in PP cells at passage 1 (*N* = 3). (**D**) Representative immunofluorescent staining of PPs in shNC, shBRD2, shBRD3, and shBRD4 groups at passage 1. The basal medium was EF6. Scale bar, 100 μm. (**E**) Total cell number of PPs treated with I-BET151, shNC, or shBRD4 (*N* = 3). The basal medium was EF6. (**F**) Bright field images of PPs in shNC and shBRD4 groups at passages 2 and 3, respectively. Scale bar, 500 μm. (**G**) Representative western blotting of BRD4 protein in PP cells treated with dBET1 (0 μM, 1 μM, and 2 μM). ACTIN

was used as a loading control. The band of BRD4 was marked by red star. $N = 3$. (**H**) Cell number of PP cells treated with DMSO, 1 μM, and 2 μM dBET1 after one week. Same number of cells were seeded in the beginning. $N = 3$. (**I**) Representative immunofluorescent staining of PPs for PDX1, NKX6.1, and nuclei. The basal medium was EF6. PP cells were treated with DMSO, 1 μM, and 2 μM dBET1 for one week respectively. Scale bar, 100 μm. All data are expressed as mean ± s.d. Statistical significance calculated using two-tailed Student's *t*-test, ns $p > 0.05$, * $p < 0.05$, ** $p < 0.01$, *** $p < 0.001$, **** $p < 0.0001$.

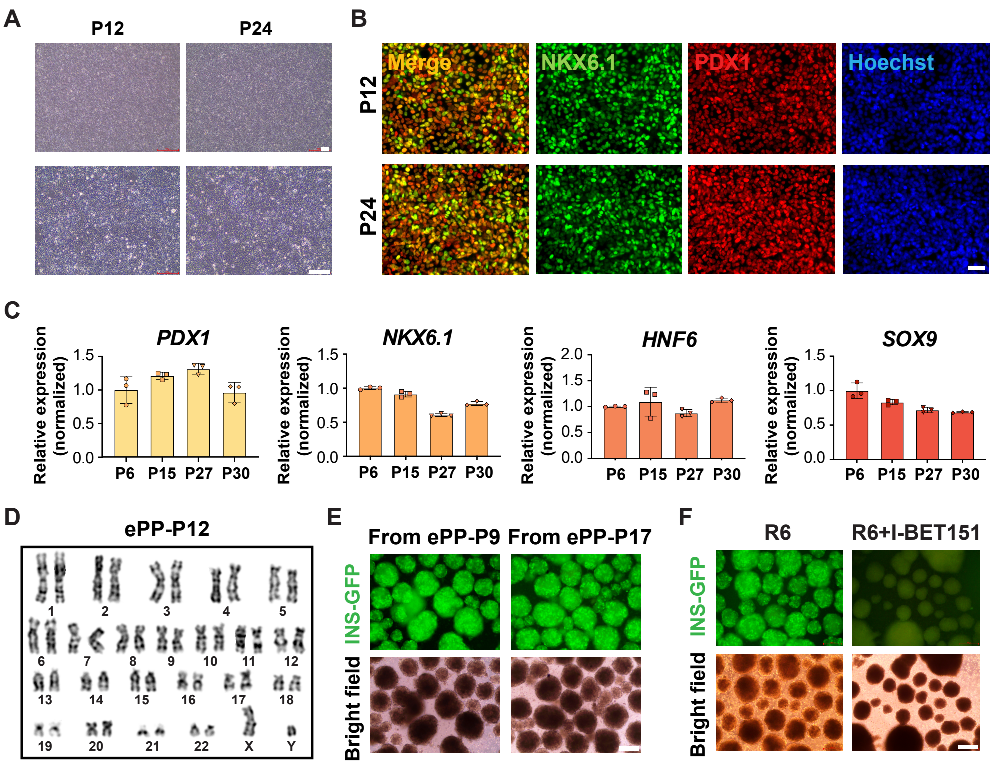

Fig. S4. Further characterization of ePPs. (**A**) Bright-field images of ePP-P12 and ePP-P24. Scale bar, 200 μm. (**B**) Representative immunofluorescent staining of ePPs at early-passage (P12) and late-passage (P24) for PDX1, NKX6.1, and nuclei (Hoechst). Scale bar, 100 μm. (**C**) RT-qPCR analysis of *PDX1*, *NKX6.1*, *HNF6*, and *SOX9* gene expression in ePP-P6, ePP-P15, ePP-P27, and ePP-P30 (N = 3). Data are expressed as mean ± s.d. (**D**) Karyotype of ePP-P12 indicates the chromatin stability of ePPs after expansion. (**E**) Representative fluorescence image of INS-GFP$^+$ ePP-β cells differentiated from ePP-P9 and ePP-P17, respectively. Scale bar, 200 μm. (**F**) Representative fluorescence image of INS-GFP$^+$ ePP-β cells indicating that I-BET151 blocked the differentiation of ePPs to ePP-β cells. R6, differentiation medium. Scale bar, 200 μm.

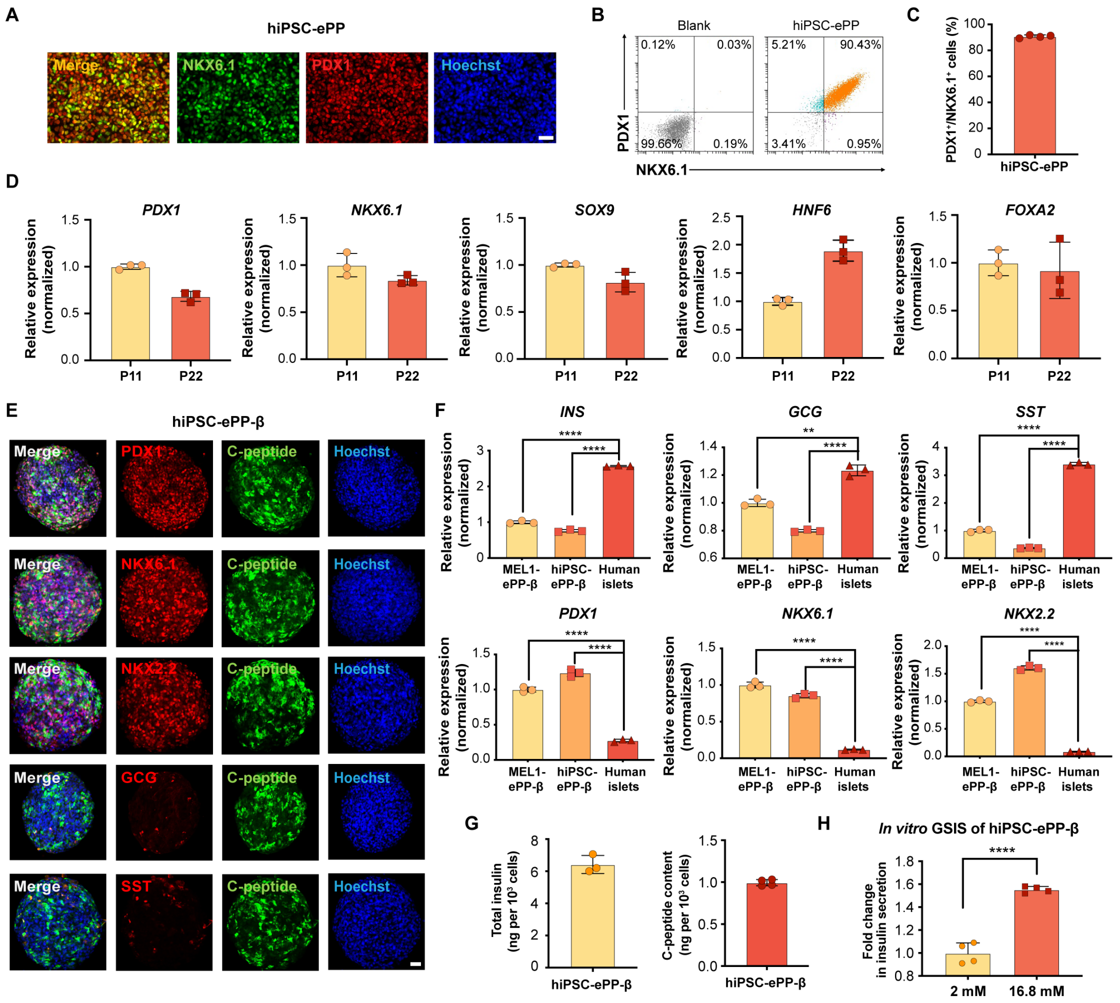

Fig. S5. Generation and characterization of ePPs and ePP-β cells from hiPSCs. (**A**) Representative immunofluorescent staining of hiPSC-derived ePPs for PDX1, NKX6.1, and nuclei (Hoechst). Scale bar, 100 μm. (**B** and **C**) Representative flow cytometry dot plots (B) and population percentages (C) of hiPSC-derived ePPs stained for PDX1 and NKX6.1 (N = 4). (**D**) RT-qPCR analysis of *PDX1*, *NKX6.1*, *SOX9*, *HNF6*, and *FOXA2* gene expression in hiPSC-derived ePPs at passage 11 (P11) and passage 22 (P22) (N = 3). (**E**) Representative immunofluorescence staining of hiPSC-derived ePP-β cells for C-peptide, PDX1, NKX6.1, NKX2.2, GCG, SST, and nuclei. Scale bar, 50 μm. (**F**) RT-qPCR analysis of *INS*, *GCG*, *SST*, *PDX1*, *NKX6.1*, and *NKX2.2* gene expression in MEL1 hESC-derived ePP-β cells, hiPSC-derived ePP-β cells, and human islets (N = 3). (**G**) Total insulin and C-peptide content in hiPSC-derived ePP-β cells. Insulin content, N = 3; C-peptide content, N = 4. (**H**) GSIS analysis of hiPSC-derived ePP-β cells in response to 2 mM and 16.8 mM glucose respectively (N = 4).

All data are expressed as mean ± s.d. Statistical significance calculated using two-tailed Student's *t*-test, ** $p < 0.01$, **** $p < 0.0001$.

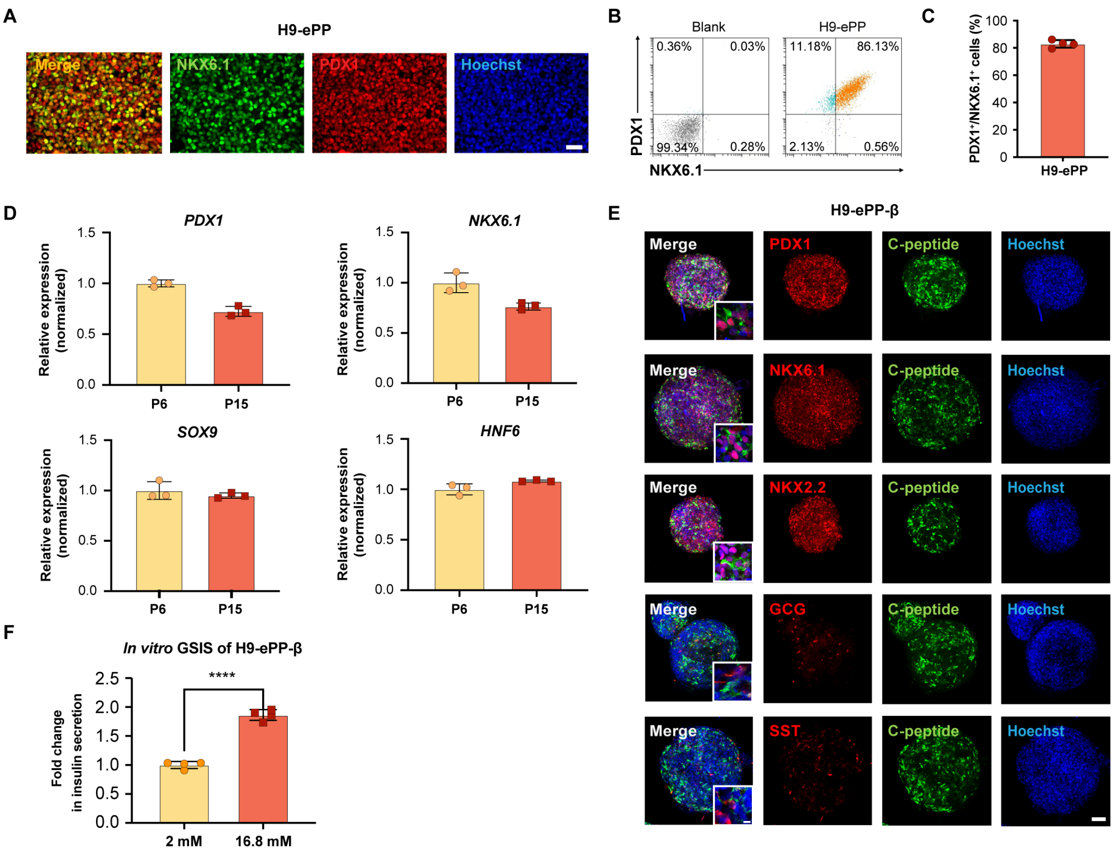

Fig. S6. Generation and characterization of ePPs and ePP-β cells from H9 hESCs. (**A**) Representative immunofluorescent staining of H9-ePPs for PDX1, NKX6.1, and nuclei (Hoechst). Scale bar, 100 μm. (**B** and **C**) Representative flow cytometry dot plots (B) and population percentages (C) of H9-ePPs stained for PDX1 and NKX6.1 (*N* = 4). (**D**) RT-qPCR analysis of *PDX1*, *NKX6.1*, *SOX9*, and *HNF6* gene expression in H9-ePPs at passage 6 (P6) and passage 15 (P15) (*N* = 3). (**E**) Representative immunofluorescence staining of H9-ePP-β cells for C-peptide, PDX1, NKX6.1, NKX2.2, GCG, SST, and nuclei. Scale bar, 100 μm (low magnification) and 10 μm (high magnification). (**F**) GSIS analysis of H9-derived ePP-β cells in response to 2 mM and 16.8 mM glucose respectively (*N* = 4). All data are expressed as mean ± s.d. Statistical significance calculated using two-tailed Student's *t*-test, **** $p < 0.0001$.

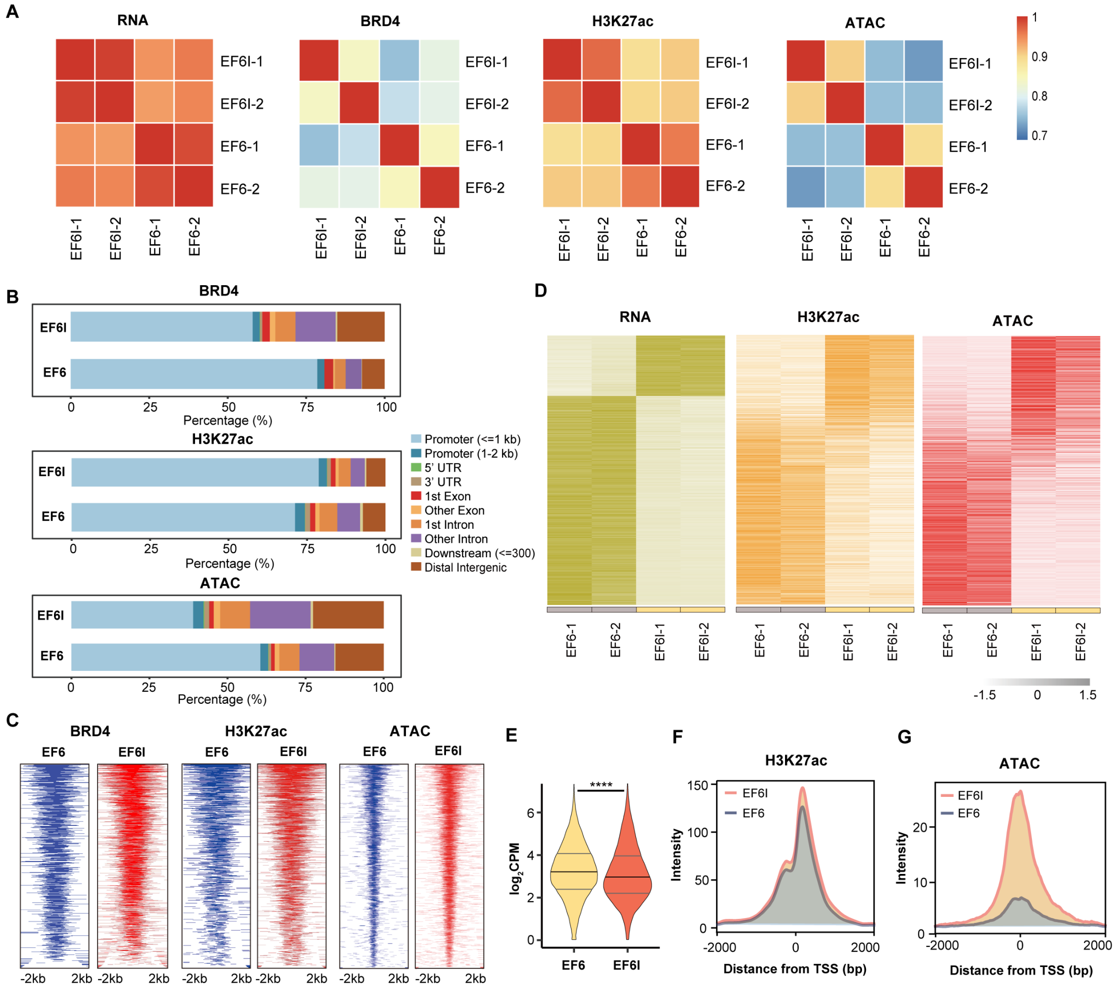

Fig. S7. Data analysis of RNA-seq, CUT&Tag, ChIP-seq, and ATAC-seq. (**A**) Correlation between sequencing samples (heatmaps of Spearman correlation coefficient). RNA-seq (left), BRD4 CUT&Tag (second), H3K27ac ChIP-seq (third), ATAC-seq (right). (**B**) Peak distribution along genome. BRD4 CUT&Tag (above), H3K27ac ChIP-seq (middle), ATAC-seq (below). (**C**) BRD4 CUT&Tag, H3K27ac ChIP-seq and ATAC-seq peak heatmaps. PPs cultured in EF6 medium (blue), PPs cultured in EF6I medium (red). (**D**) Epigenome and transcriptome changes for 2829 differentially expressed genes in PPs treated with or without I-BET151. RNA-seq (olivine), H3K27ac ChIP-seq (orange), ATAC-seq (red). (**E**) Violin plot of BRD4-binding peak abundance in PP cells cultured in EF6 or EF6I conditions. The upper and lower quartiles are indicated in grey. The median is indicated in black. Statistical significance calculated using Kolmogorov-Smirnov test, **** $p < 0.0001$. (**F**) H3K27ac peak intensities in PPs treated with or without I-BET151. (**G**) Chromatin accessibility at sites significantly increased by I-BET151.

Table S1. Key resource table

Reagent or Resource	Source	Identifier
**Antibodies**		
goat anti-m/rPDX1	R&D SYSTEMS	Cat#AF2517
goat anti-hPDX1	R&D SYSTEMS	Cat#AF2419
mouse anti-NKX6.1	DSHB	Cat#F55A12
mouse anti-SOX9	Santa cruz	Cat#sc-166505
rabbit anti-FOXA2	Sigma-Aldrich	Cat#07-633
rabbit anti-Ki67	Abcam	Cat#ab15580
mouse anti-NKX2.2	DSHB	Cat#74.5A5
goat anti-Somatostatin (SST)	Santa cruz	Cat#sc-7819
goat anti-Glucagon (GCG)	Santa cruz	Cat#sc-7780
rat anti-C-peptide	DSHB	Cat#GN-ID4
rabbit anti-C-peptide	Abcam	Cat#ab14181
rabbit anti-MAFA	Cell Signaling Technology	Cat#D2Z6N
rabbit anti-H3K27ac	Abcam	Cat#ab4729
rabbit anti-BRD4	Abcam	Cat#ab128874
mouse anti-β-ACTIN	Sungenebiotech	Cat#400-6210003
Alexa Fluor 488 donkey anti-mouse IgG (H+L)	Invitrogen	Cat#A-21202
Alexa Fluor 555 donkey anti-mouse IgG (H+L)	Invitrogen	Cat#A-31570
Alexa Fluor 555 donkey anti-rabbit IgG (H+L)	Invitrogen	Cat#A-31572
Alexa Fluor 555 donkey anti-goat IgG (H+L)	Invitrogen	Cat#A-21432
Alexa Fluor 488 donkey anti-rabbit IgG (H+L)	Invitrogen	Cat#A-21206
Alexa Fluor 647 donkey anti-goat IgG (H+L)	Invitrogen	Cat#A-21447
Alexa Fluor 488 donkey anti-rat IgG (H+L)	Invitrogen	Cat#A-21208
HRP-goat anti-mouse IgG	EARTHOX	Cat#E030110-01
HRP-goat anti-rabbit IgG	EARTHOX	Cat#E030120-01
**Bacterial Strains**		
DH5α	ANGYUBIO	Cat#AYBIO-G6016
**Chemicals, Peptides, and Recombinant Proteins**		
I-BET151	APE×BIO	Cat#B1500
dBET1	TargetMol	Cat#T4495
Polybrene	Santa cruz	Cat#sc-134220
CHIR99021	TargetMol	Cat#T2310
616452	TargetMol	Cat#T6337
3,3’,5-Triiodo-l-thyronine sodium salt (T3)	Sigma	Cat#T6397
A83-01	TargetMol	Cat#T10442
TTNPB	Sigma	Cat#T3757

Forskolin	TargetMol	Cat#T2939
Vitamin C	Sigma-Aldrich	Cat#A8960-5G
NAC	Sigma-Aldrich	Cat#A9165
(+)-JQ1	MedChem Express	Cat#HY-13030
(-)-JQ1	MedChem Express	Cat#HY-13030A
LDN-193189	Tocris	Cat#6053
Compound E	MedChem Express	Cat#HY-14176
Thiazovivin	TargetMol	Cat#T2155
Trolox	Sigma	Cat#238813
Heparin	Sigma	Cat#H3149
Zinc sulfate	Sigma	Cat#Z0251
Human bFGF	Peprotech	Cat#100-18B
Human KGF	Peprotech	Cat#100-19
Human EGF	Peprotech	Cat#AF-100-15
Human/Murine/Rat Activin A	Peprotech	Cat#120-14P
Human Heregulinβ-1	Peprotech	Cat#100-03
DMEM basic	Gibco	Cat#C11995500CP
DMEM/F12	Gibco	Cat#11320033
RPMI 1640	Gibco	Cat#31870082
B-27 Supplement (50X)	Gibco	Cat#0080085SA
BSA	Yeasen	Cat#36101ES25
Penicillin-Streptomycin	Gibco	Cat#15140-122
Non-essential amino acids	Gibco	Cat#11140-050
2-Mercaptoethanol	Sigma-Aldrich	Cat#M3148
Fetal bovine serum	Gibco	Cat#10270-106
Knockout serum replacement	Gibco	Cat#10828-028
Insulin-Transferrin-Selenium-Ethanolamine (ITS-X)	Gibco	Cat#51500056
StemPro Accutase Cell Dissociation Reagent	Gibco	Cat#A1110501
**Assay Kits**		
DNA Clean & Concentrator-5	ZYMO	Cat#D4014
Quick-RNA MiniPrep Kit	ZYMO	Car#R1054
Magna ChIP A/G One-Day Chromatin Immunoprecipitation Kit	Millipore	Cat#17-10086
VAHTS Universal DNA Library Prep Kit for Illumica V2	Vazyme	Cat#ND607
VAHTS DNA Adapters Set1 for Illumina	Vazyme	Cat#N801-01
TruePrep DNA Library Prep Kit V2 for Illumina	Vazyme	Cat#TD501
Qubit dsDNA BR Assay Kit	Invitrogen	Cat#Q32850
TruePrep Index Kit V2 for Illumina	Vazyme	Cat#TD202
Endo-Free Plasmid Mini Kit	Omega	Cat#D6950

Agarose Gel DNA Extraction Kit	Easydo	Cat#0103050
PrimeScript RT Master Mix	Takara	Cat#RR036A
TB Green Premix Ex Taq II Kit	Takara	Cat#RR820A
Human Insulin Immunoassay Kit	EZassay	Cat#HM200
Human C-peptide Ultrasensitive ELISA Kit	Mercodia	Cat#10-1141-01
**Deposited Data**		
All sequencing data	This paper	GEO: GSE156712
**Oligonucleotides**		
shRNA: NC (forward): 5'-CCGGCAACAAGATGAAGAGCACCAACTCGAGTTGGTGCTCTTCATCTTGTTGTTTTTG-3'	N/A	N/A
shRNA: NC (reverse): 5'-AATTCAAAAACAACAAGATGAAGAGCACCAACTCGAGTTGGTGCTCTTCATCTTGTTG -3'	N/A	N/A
shRNA: *BRD2* (forward): 5'-CCGGCCGGAAGCCCTACACCATTAACTCGAGTTAATGGTGTAGGGCTTCCGGTTTTTG -3'	Sigma	N/A
shRNA: *BRD2* (reverse): 5'-AATTCAAAAACCGGAAGCCCTACACCATTAACTCGAGTTAATGGTGTAGGGCTTCCGG -3'	Sigma	N/A
shRNA: *BRD3* (forward): 5'-CCGGGTGAGATTCGTACCGAAGAACCTCGAGGTTCTTCGGTACGAATCTCACTTTTTTG -3'	Sigma	N/A
shRNA: *BRD3* (reverse): 5'-AATTCAAAAAAGTGAGATTCGTACCGAAGAACCTCGAGGTTCTTCGGTACGAATCTCAC -3'	Sigma	N/A
shRNA: *BRD4* (forward): 5'-CCGGCCTGGAGATGACATAGTCTTACTCGAGTAAGACTATGTCATCTCCAGGTTTTTG-3'	Sigma	N/A
shRNA: *BRD4* (reverse): 5'-AATTCAAAAACCTGGAGATGACATAGTCTTACTCGAGTAAGACTATGTCATCTCCAGG-3'	Sigma	N/A
Primers for RT-qPCR, see Table S2	N/A	N/A
**Software and Algorithms**		
GraphPad Prism	GraphPad Software	http://www.graphpad.com

SnapGene	N/A	https://www.snapgene.com/
Rstudio	N/A	https://www.rstudio.com/
Wave	Agilent	https://www.agilent.com/zh-cn/products/cell-analysis/software-download-for-wave-desktop
HISAT2 (v2.1.0)	N/A	http://daehwankimlab.github.io/hisat2/
stringtie (v2.0)	N/A	http://ccb.jhu.edu/software/stringtie/
Bowtie2 (v2.2.5)	N/A	http://bowtie-bio.sourceforge.net/bowtie2
MACS2 (v2.1.2)	N/A	https://pypi.org/project/MACS2/
IGV (v2.6.3)	N/A	http://www.igv.org/
picard-tools (v2.20.5)	N/A	http://broadinstitute.github.io/picard/
HOMER (v4.11)	N/A	http://homer.ucsd.edu/homer/
deepTools (v3.4.3)	N/A	https://deeptools.readthedocs.io/en/develop/
samtools (v1.9)	N/A	http://samtools.sourceforge.net/
sambamba (v0.7.1)	N/A	https://github.com/biod/sambamba

Table S2. Primer sequences

Primer	Sequence
GAPDH-F	TGCACCACCAACTGCTTAGC
GAPDH-R	GGCATGGACTGTGGTCATGAG
FOXA2-F	GGGAGCGGTGAAGATGGA
FOXA2-R	TCATGTTGCTCACGGAGGAGTA
HNF6-F	ATGTCCAGCGTCGAACTCTAC
HNF6-R	TGCTTTGGTACAAGTGCTTGAT
SOX9-F	AGCTCTGGAGACTTCTGAACGAGAG
SOX9-R	CGTTCTTCACCGACTTCCTCCGC
PDX1-F	CCTTTCCCATGGATGAAGTC
PDX1-R	GAACTCCTTCTCCAGCTCTA

NKX6.1-F	TCAACAGCTGCGTGATTTTC
NKX6.1-R	CCAAGAAGAAGCAGGACTCG
SOX17-F	ATTTCCTCGGTGGTGTCC
SOX17-R	CCAAACTGTTCAAGTGGCAGA
OCT4-F	GAGAAGGAGAAGCTGGAGCA
OCT4-R	AATAGAACCCCCAGGGTGAG
NANOG-F	GATTTGTGGGCCTGAAGAAA
NANOG-R	CAGATCCATGGAGGAAGGAA
BRD2-F	GGAAACATCAGTTCGCATGGC
BRD2-R	CACTCTGAAGCAGCCCAATAA
BRD3-F	ATCACTGCAAACGTCACGTC
BRD3-R	CCTGCTTGGGGTCTGACAAC
BRD4-F	GAGCTACCCACAGAAGAAACC
BRD4-R	GAGTCGATGCTTGAGTTGTGTT
mtDNA 16S rRNA-F	GCCTTCCCCCGTAAATGATA
mtDNA 16S rRNA-R	TTATGCGATTACCGGGCTCT
β2M-F	TGCTGTCTCCATGTTTGATGTATCT
β2M-R	TCTCTGCTCCCCACCTCTAAGT

Table S3. Information of small molecules in the chemical library

No.	Full Name	Function(s)	Concentration (μM)
1	CHIR99021	GSK3 inhibition	3
2	E616452	TGFβ inhibition	10
3	Thiazovivin	ROCK inhibition	1
4	Vitamin C	Antioxidant	250
5	Parnate	LSD1 histone demethylase inhibition	1
6	Forskolin	PKA activation	10
7	Valproic acid	Histone deacetylase inhibition	500
8	AM580	RAR activation	0.5
9	EPZ004777	Histone methyltransferase inhibition	1
10	A83-01	TGFβ inhibition	1
11	SB431542	TGFβ inhibition	10
12	Sodium butyrate	Histone deacetylase inhibition	100
13	BIX01294	Histone methyltransferase inhibition	0.1
14	RG108	DNA methyltransferase inhibition	1
15	Kenpaullone	Dual inhibition of CDK and GSK3β	1
16	Bayk8644	L-type Ca^{2+} channel activation	1
17	SP600125	JNK inhibition	1
18	SB203580	p38 MAPK inhibition	1
19	LPA	Metabolite	1
20	Rolipram	PDE4 inhibition	1

21	SC1	Dual inhibition of ERK1 and Ras GTPase	0.1
22	Dexamethasone	Glucocorticoid receptor agonist	1
23	SB590885	B-Raf inhibition	1
24	WH-4-023	Lck/Src inhibition	1
25	Go 6983	PKC inhibition	1
26	SMER28	Positive regulation of autophagy	1
27	Pentamine	Ganglionic blocker	0.1
28	BAF312	S1P receptor agonist	1
29	TTNPB	RAR activation	1
30	SGC0946	DOT1L methyltransferase inhibition	1
31	PD0325901	MEK inhibition	0.1
32	5-Azacytidine	DNA methyltransferase inhibition	0.5
33	Retinoic acid	RAR activation	1
34	GDC-0449	Hedgehog inhibition	0.1
35	2-Hydroxyglutarate	Metabolite	100
36	dm-αKG	Metabolite	100
37	Trolox	ROS inhibition	10
38	IWR-1	Wnt inhibition	1
39	Compound E	Notch inhibition	0.1
40	R428	TAM receptor inhibition	1
41	N-acetyl cysteine	Antioxidant	1,000
42	LDE225	Hedgehog inhibition	0.1
43	SU5402	Dual inhibition of FGF and VEGF receptors	1
44	Isx9	Ca^{2+} channel activation	1
45	L755507	Adrenergic receptor activation	0.1
46	Brefeldin A	ATPase inhibition	0.1
47	Azidothymidine	DNA polymerase inhibition	0.1
48	Trifluridine	DNA/RNA synthesis inhibition	0.1
49	SCR7	DNA/RNA synthesis inhibition	0.1
50	Sal003	eIF-2α phosphatase inhibition	1
51	KY02111	Wnt/beta-catenin inhibition	1
52	SW033291	Dehydrogenase inhibition	1
53	SR202	PPARγ inhibition	1
54	SB202190	p38 MAPK inhibition	1
55	UNC0638	G9a/GLP histone methyltransferase inhibition	1
56	Methylthioadenosine	Metabolite	1
57	A-366	G9a/GLP histone methyltransferase inhibition	1
58	UNC0642	G9a/GLP histone methyltransferase inhibition	1
59	EPZ015666	PRMT5 histone methyltransferase inhibition	1
60	EPZ031686	SMYD3 histone methyltransferase inhibition	1
61	EPZ-5676	DOT1L histone methyltransferase inhibition	1
62	EPZ-6438	EZH2 histone methyltransferase inhibition	1
63	GSK 126	EZH2 histone methyltransferase inhibition	1

64	GSK 343	EZH2 histone methyltransferase inhibition	1
65	PFI-2	SETD7 histone methyltransferase inhibition	1
66	SGC707	PRMT3 histone methyltransferase inhibition	1
67	UNC1999	EZH2 histone methyltransferase inhibition	1
68	StemRegenin-1	Aryl hydrocarbon receptor inhibition	1
69	I-BET151	BET bromodomain inhibition	1
70	Necrostatin-1	Necrosis inhibition	1
71	17β-estradiol	Estrogen	1
72	P7C3	NAMPT activation	1
73	Lithium chloride	GSK3 inhibition	1,000
74	AS8351	Epigenetic modulation	1
75	Purmorphamine	Hedgehog activation	1
76	Liproxstatin-1	Ferroptosis inhibition	1
77	D4476	CK inhibition	1
78	PP1	Src inhibition	1
79	PP2	Src inhibition	1
80	Zebularine	DNA methyltransferase inhibition	1
81	IWP-2	Wnt inhibition	1
82	Reversine	Aurora kinase inhibition	1
83	Dasatinib	Bcr-Abl and Src family inhibition	1
84	AMI-5	Histone arginine methyltransferase inhibition	1
85	Berberine	Anti-microbial	1
86	Rapamycin	mTOR inhibition	0.1
87	Nabumetone	COX inhibition	1
88	SR11237	RXR activation	1
89	Arctigenin	Antioxidant	1
90	TUDCA	Endoplasmic reticulum stress inhibition	1
91	Capsaicin	TRPV1 channel activation	1
92	Bay11-7082	NF-κB inhibition	1
93	Luteolin	NF-κB inhibition	1
94	IOX1	Histone demethylase inhibition	1
95	GSK 4716	ERRβ/γ activation	1
96	Azaserine	Glutamine amide transferase inhibition	1
97	AEG3482	JNK inhibition	1
98	THIQ	Melanocortin-4 receptor activation	1
99	Nilotinib	Bcr-Abl tyrosine kinase inhibition	1
100	Idebenone	ROS inhibition	1
101	CD3254	RXR activation	1
102	TBZ	Vesicular monoamine transporter 2 inhibition	1
103	CD437	RAR activation	1
104	SR12813	Pregnane X receptor activation	1
105	DY131	ERRβ/γ activation	1
106	NECA	Adenosine receptor activation	1

107	TPB	AChR activation	0.2
108	T3	TRβ1 activation	1
109	6-BIO	GSK3 inhibition	0.1
110	TSA	Histone deacetylases inhibition	0.01
111	25-HC	HMG-CoA reductase inhibition	1
112	FH1	Others	1
113	FPH1	Others	1
114	Pyrintegrin	Others	1
115	DMF	Metabolite	10
116	Meclizine	Histamine receptor inhibition	1
117	Pifithrin-α	p53 inhibition	1
118	Bay11-7085	IκBα phosphorylation inhibition	1
119	CsA	Calcineurin inhibition	0.1
120	STF31	Glucose transporter 1 inhibition	0.1
121	SR12813	Pregnane X receptor activation	1
122	Betulinic acid	Topoisomerase inhibition	1
123	Pitstop2	Clathrin-mediated endocytosis inhibition	1
124	BML-260	JSP-1 inhibition	1
125	T0901317	LXR and FXR activation	1
126	3-Aminobenzoic acid	PARP inhibition	1
127	DON	Glutaminase inhibition	1
128	BIX00089	Others	1
129	Rif	Homologous recombination inhibition	1
130	GW501516	PPARβ activation	1
131	AICAR	AMPK activation	1
132	Metformin	AMPK activation	1
133	SR9009	REV-ERBα/β activation	1
134	OAC1	Epigenetic modulation	1
135	OAC2	Epigenetic modulation	1
136	OAC3	Epigenetic modulation	1
137	IC261	CK1 inhibition	1
138	UNC0646	G9a/GLP histone methyltransferase inhibition	1
139	UNC0631	G9a histone methyltransferase inhibition	1
140	EGCG	Telomerase and DNMT inhibition	1
141	AOA	Aminotransferase inhibition	1
142	db-cAMP	PKA activation	1
143	GSK8470	NR5A2 activation	1
144	6-AN	$NADP^+$-dependent enzyme inhibition	0.1
145	BX912	PDK inhibition	1
146	Rotenone	p53 activator	0.01
147	PDBu	PKC activation	0.1
148	PGE2	Metabolite	1
149	Def	Others	1

150	Wortmannin	PI3K inhibition	0.1
151	DM-PGE2	Endogenous metabolite	2.6
152	CAPE	NF-κB inhibition	1
153	Triptolide	NF-κB inhibition	1
154	DNP	Others	1
155	Sacrosine	GlyT inhibition	100
156	Atrazine	Endocrine inhibition	1
157	PDTC	NF-κB inhibition	1
158	Quercetin	Mitochondrial ATPase and PDE inhibition	1
159	CD1530	RARγ receptor activation	1
160	Piceatannol	NF-κB inhibition	1
161	PS48	PDK1 activation	1
162	JNKiII	JNK inhibition	1
163	Oligomycin	ATP synthase inhibition	0.01
164	ILV	PKC activation	1
165	Z-VAD-FMK	Caspase inhibition	1
166	SCD1i	SCD1 inhibition	0.1
167	LAI	Others	1
168	RWJ-60475	Phosphatase inhibition	1
169	6-AN	$NADP^+$-dependent enzyme inhibition	1
170	Oxaloacetic acid	Metabolite	100
171	Pregnenolone	AChR activation	1
172	5-IT	Adenosine kinase inhibition	1
173	Dorsomorphin	AMPK inhibition	0.1
174	4-PBA	ER inhibition	100
175	UNC-01	PKC inhibition	0.1
176	Ciclopirox	Anti-inflammatory	1
177	17-AAG	Hsp90 inhibition	1
178	BisI	PKC inhibition	1
179	QHS-1	Others	1
180	QHS-2	Others	1
181	QHS-3	Others	1
182	QHS-4	Others	1
183	QHS-5	Others	1
184	QHS-6	Others	1
185	QHS-7	Others	1
186	QHS-8	Others	1
187	QHS-9	Others	1
188	PDE-10	Others	1
189	LMP-11	Others	1
190	LMP-12	Others	1
191	LMP-13	Others	1
192	DFC-14	Others	1

193	DFC-15	Others	1
194	DFC-16	Others	1
195	DFC-17	Others	1
196	DFC-18	Others	1
197	DFC-19	Others	1
198	DFC-20	Others	1
199	DFC-21	Others	1
200	DFC-22	Others	1
201	DFC-23	Others	1
202	DFC-24	Others	1
203	DFC-25	Others	1
